# The vascular nature of lung‐resident mesenchymal stem cells

**DOI:** 10.1002/sctm.20-0191

**Published:** 2020-08-24

**Authors:** Jennifer Steens, Lea Klar, Christine Hansel, Alexis Slama, Thomas Hager, Verena Jendrossek, Clemens Aigner, Diana Klein

**Affiliations:** ^1^ Institute of Cell Biology (Cancer Research) University of Duisburg‐Essen, University Hospital Essen Germany; ^2^ Department of Thoracic Surgery and Surgical Endoscopy Ruhrlandklinik‐University Clinic Essen Essen Germany; ^3^ Institute of Pathology University Clinic Essen, University of Duisburg‐Essen Essen Germany

**Keywords:** adventitia, lung cancer, mesenchymal stem cells, NSCLC, stem cell niche, vascular niche

## Abstract

Human lungs bear their own reservoir of endogenous mesenchymal stem cells (MSCs). Although described as located perivascular, the cellular identity of primary lung MSCs remains elusive. Here we investigated the vascular nature of lung‐resident MSCs (LR‐MSCs) using healthy human lung tissue. LR‐MSCs predominately reside within the vascular stem cell niche, the so‐called vasculogenic zone of adult lung arteries. Primary LR‐MSCs isolated from normal human lung tissue showed typical MSC characteristics in vitro and were phenotypically and functionally indistinguishable from MSCs derived from the vascular wall of adult human blood vessels (VW‐MSCs). Moreover, LR‐MSCs expressed the VW‐MSC‐specific HOX code a characteristic to discriminate VW‐MSCs from phenotypical similar cells. Thus, LR‐MSC should be considered as VW‐MSCs. Immunofluorescent analyses of non‐small lung cancer (NSCLC) specimen further confirmed the vascular adventitia as stem cell niche for LR‐MSCs, and revealed their mobilization and activation in NSCLC progression. These findings have implications for understanding the role of MSC in normal lung physiology and pulmonary diseases, as well as for the rational design of additional therapeutic approaches.

## Graphical abstract

Although the precise stem cell niche(s) of lung‐resident mesenchymal stem cells (LR‐MSCs) remain(s) elusive, lung injury following a pathological trigger might at least be partially mediated by endogenous MSCs. Here we show that LR‐MSCs predominately reside within the vascular stem cell niche, the so‐called vasculogenic zone of adult lung arteries, and reveal their mobilization and activation in lung cancer progression.

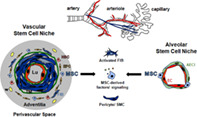

**Significance statement**

Although therapeutically applied mesenchymal stem cells (MSCs) turned out to be a valuable therapeutic option for the prevention and regeneration of lung diseases because of their relatively easy availability, multipotent differentiation capacities, and immunomodulatory effects, the role of endogenous lung MSCs is less clear. This article reports that lung‐resident MSCs were not only tissue‐resident, as revealed by their localization within the adventitia of lung blood vessels, but also tissue‐specific and in particular showed tissue specificity of the vessel wall. As a potential cellular origin and/or as mesenchymal cells of the tumor stroma, these cells critically affect lung cancer progression.

## 1 INTRODUCTION

Tissue‐resident stem cells are responsible for the tissue's turnover, renewal, and repair. Among these adult stem cells, mesenchymal stem cells, also designated as mesenchymal stromal cells (MSCs), are important regulators of tissue homeostasis. Herein, tissue‐resident MSCs harbor the potential to replace defunct cells or secrete cytokines locally and thus support repair and healing processes of affected tissues. This regenerative potential together with their anti‐inflammatory properties have paved the way for using both autologous and allogeneic MSCs for cell‐based therapies in order to treat, for example, acute tissue injury syndromes, chronic degenerative disorders, and inflammatory disease.^1^, ^2^ Different tissue sources of MSCs have even been successfully used in preclinical studies and in clinical trials for the treatment of severe lung diseases. However, less is known about tissue‐resident MSCs in adult human lungs. Although it is clear that human lungs bear their own reservoir of endogenous MSCs (lung‐resident MSCs [LR‐MSCs]) and that LR‐MSCs contribute to the maintenance of lung integrity, there is less clarity what resident lung MSCs exactly are and what their role and function in lung homeostasis as well as diseased states may be.^3^, ^4^, ^5^, ^6^ In particular, it remains elusive where exactly to find residing MSCs within the human lung tissue.^7^ Moreover, following a pathological trigger, LR‐MSCs seem not to appear sufficient for lung tissue protection or repair. LR‐MSCs could even contribute to the development of pulmonary diseases, for example, via differentiation to myofibroblasts, which have been proposed as the main source of extracellular matrix within impaired lungs, and/or by modulating cancer progression, because of their ability to migrate toward primary tumors and metastatic sites.^8^ Protecting the endogenous stem cell pool or rescuing deficient endogenous lung MSCs by applying exogenous MSCs will limit lung injury or foster lung tissue regeneration.^9^, ^10^

The identification and subsequent isolation of lung stem cells and particularly LR‐MSCs remains a major challenge because of the absence of markers that are uniquely expressed by lung stem cells. MSC isolations in general, were found to be quite heterogeneous although phenotypically these cells were highly similar, and thus meet all the criteria as defined by the International Society of Cell Therapy (ISCT).^11^, ^12^ The minimal criteria markers for defining in vitro expanded MSC are plastic adherence; CD73, CD90, and CD105 expressions while lacking the expression of hematopoietic and endothelial markers; the capability of in vitro differentiation into adipocyte, chondrocyte, and osteoblast lineages.^11^, ^12^ Multiple protocols exist to isolate LR‐MSCs bearing those classical MSC properties. Besides, various cell separation strategies using cell surface antigens and “side population” characteristics, that is the capability of stem cells to efflux the fluorescent DNA‐binding dye Hoechst 33342 based on the ATP‐binding cassette transporters, were used to characterize and isolate pulmonary side population phenotypes. Well‐defined culture conditions using appropriate media for additional stem cell‐type selection have further been used to investigate the lungs rare populations of multipotent and additionally mesenchymal endogenous stem cells. But again, the precise localization of LR‐MSCs within the lung tissue remains elusive.

Adult human arteries harbor a stem cells niche, the so‐called vasculogenic zone, where different stem and progenitor cells reside (including MSCs) that play decisive roles in the development, normal physiology, and pathophysiology of many diseases.^13^, ^14^, ^15^, ^16^, ^17^, ^18^ These vascular stem cells here comprise an important source of all types of vascular cells needed to ensure the processes of neovascularization, vascular remodeling, as well as blood vessel repair.^19^, ^20^, ^21^, ^22^ Here we investigated the vascular wall as stem cell niche of LR‐MSCs. We hypothesize that LR‐MSCs might originate from the vessel wall. Immunohistochemistry as well as co‐immunofluorescence analyses were used to confirm the vascular adventitia as stem cell niche for LR‐MSCs in normal lung tissue as well as diseased lung tissue in particular in non‐small cell lung cancer (NSCLC) tissue. LR‐MSCs were further isolated from healthy human lung tissue and compared to MSCs derived from the wall of adult human blood vessels (VW‐MSCs). LR‐MSCs were not only tissue‐resident but also tissue‐specific and in particular showed tissue specificity of the vessel wall. Thus, at least in the lung, the distribution of MSCs throughout the post‐natal organism could be related to their existence in a vascular niche. These findings have implications for understanding role of MSC in normal lung physiology and pulmonary diseases, as well as for the rational design of additional in vivo therapeutic approaches.

## 2 MATERIALS AND METHODS

### 2.1 Tissue material

Normal lung tissue samples were obtained during surgery according to local ethical and biohazard regulations and provided from the Department of Thoracic Surgery and Surgical Endoscopy, Ruhrlandklinik, University Hospital Essen for our institute. All experiments were performed in strict accordance with local guidelines and regulations. Written informed consent (17‐7454‐BO) was obtained from the Ethikkommission of the University Medical Faculty, Essen, Germany.^23^ Samples were collected in close cooperation with the local biobank (Westdeutsche Biobank Essen). Resected tissue specimens were processed for pathological diagnostic routine in agreement with institutional standards and diagnoses were made based on current WHO and updated ISUP criteria. Human tissue samples were analyzed anonymously.

### 2.2 LR‐MSCs isolation

Adventitial MSCs were isolated from human internal thoracic arteries (fragments as previously described).^18^, ^24^, ^25^, ^26^ The protocol was adapted for the isolation of LR‐MSCs. Briefly, after several washes in PBS (containing P/S), normal human lung tissues were mechanically minced and dissociated for 20‐30 minutes at 37°C in OptiMEM I medium (GIBCO) containing 0.2% type 2‐collagenase (Worthington, Lakewood) and 5 U/mL elastase (Sigma). On dissociation, cells were washed twice in PBS containing 5% FCS (300*g*, 10 minutes, 4°C). Cellular suspensions were passed through 70 mm pore size filters. Primary LR‐MSCs were cultivated on plastic cell culture plates using complete human MSC‐GM media (PromoCell, Heidelberg, Germany). Medium was removed 24 hours after initial plating, nonadherent cells were washed away and fresh medium was replaced.

### 2.3 RNA isolation, cDNA synthesis, and quantitative real‐time RT‐PCR analysis

For RNA isolation, cells were lysed directly in culture dishes as previously described.^24^, ^27^ RNA was isolated using RNeasy Mini Kit and cDNA synthesis with integrated genomic DNA removal was performed using QuantiTect Reverse Transcription (Qiagen, Hilden, Germany) according to the manufacturer's instructions. Real‐time RT‐PCR analysis was carried out using specific deoxy‐oligonucleotide primers.^24^, ^25^

### 2.4 Flow cytometry

For flow cytometric analysis, cells were treated with trypsin/EDTA (Life Technologies) to generate single‐cell suspensions. For each staining reaction, 1 × 10^5^ cells were incubated with fluorochrome‐coupled antibody (antigen‐specific or isotype control) in 100‐200 μL FACS buffer (5% [vol/vol] FBS in PBS) for 20 minutes at 4°C.^25^, ^28^ Cells were washed twice and subsequently re‐suspended in 200 μL FACS buffer and analyzed on a FACS Aria III (Becton Dickinson) using the FACSDiva software (Becton Dickinson).

### 2.5 Colony‐forming unit assay

The cells were plated at low densities (100‐1000 cells per well; triplicates). The medium was changed every 2 days. After 10 days' culture, the cells were washed with PBS, fixed with 4% (wt/vol) paraformaldehyde, and subsequently stained with 0.05% Coomassie brilliant blue. Then the colonies (≥50 cells/colony) were counted. The survival curves were established by plotting the log of the surviving fraction.^9^, ^25^

### 2.6 Trilineage differentiation assay

Differentiation of cultivated MSCs into adipocytes, chondrocytes, and osteocytes was done using ready‐to‐use differentiation media from Lonza (hMSC Differentiation BulletKit‐Adipogenic, PT‐3004; Chondrogenic, PT‐3003; Osteogenic, PT‐3002) according to the manufacturer's instructions. Adipogenic differentiation was verified using oil red staining, chondrogenic differentiation was verified using collagen type II antibody (Santa Cruz) and immunohistochemistry or Alcian blue staining solution (1% wt/vol Alcian blue in acetic acid, pH 2.5), and osteogenic differentiation was verified using NBT (nitro‐blue tetrazolium chloride) and BCIP (5‐bromo‐4‐chloro‐3′‐indolyphosphate p‐toluidine salt) staining (Sigma) for alkaline phosphatase activity.^24^, ^25^, ^28^

### 2.7 Mixed lymphocyte reaction

For mixed lymphocyte reactions, LR‐MSCs as well as VW‐MSCs were plated in 96‐well plates (5000 cells per well). After adherence, the plates were left untreated or irradiated with 10 Gy.^25^, ^28^ After additional 24 hours, medium was exchanged and lymphocytes of different lines were added (10 000 cells per well): human lymphoma cells (MOLT17 [ACC 36] T‐cell leukemia, DoHH2 non‐Hodgkin's B‐cell lymphoma cells [both from Leibniz Institute DSMZ‐German Collection of Microorganisms and Cell Cultures, Braunschweig, Germany], Jurkat, Clone E6‐1 peripheral blood T lymphocyte, U937 myeloid lineage histiocytic lymphoma [both ATCC/LGC Standards]). After additional 24 and 96 hours of coculture, cell proliferation was determined using the WST‐1 colorimetric assay. Values were compared to that one obtained from single lymphocyte cultures.

### 2.8 Immunohistochemistry and immunofluorescence

Paraffin‐embedded tissue sections were hydrated using a descending alcohol series, incubated for 10 to 20 minutes in target retrieval solution (DAKO, Glostrup, Denmark). Sections were incubated with primary antibodies in blocking solution (2% FCS/PBS) over night at 4°C. Antigens were detected with respective fluorescence‐conjugated secondary antibodies (1/500) or with horseradish peroxidase‐conjugated secondary antibodies (1/500) and DAB staining.^25^, ^28^

### 2.9 Spheroid culture and colony‐forming unit assay of lung cancer cells

For spheroid culture, NCI‐H460 (H460) cells were cultured alone or in combination with LR‐MSCs and VW‐MSCs in hanging drops for 24 hours (ratio 1/1). Then, spheroids were plated in normal growth medium (NGM) with growth‐factor‐reduced Matrigel (Corning, New York) (dilution: 1/2) and analyzed additional 48 hours later as previously described.^29^ For colony‐forming unit (CFU), H460 cells were plated (triplicates) at low densities (100‐1000 cells), irradiated and subsequently incubated with supernatants/conditioned media of LR‐MSCs and VW‐MSCs for additional 10 days. Complete protocols are provided in the Supplemental Information.

### 2.10 The Kaplan‐Meier plotter

The prognostic significance of the mRNA expression of indicated genes in NSCLC was evaluated using the Kaplan‐Meier plotter (www.kmplot.com), an online database including gene expression data and clinical data of lung cancer.^30^ Therefore, HOX code genes as well as the CD44 gene were uploaded into the database respectively to obtain the Kaplan‐Meier survival plots, in which the number‐at‐risk was shown below the main plot. Log rank *P*‐value and hazard ratio (HR) with 95% confidence intervals were calculated and displayed on the webpage.^31^

### 2.11 Statistical analysis

If not otherwise indicated (n = biological replicates), data were obtained from at least from three independent experiments. Data analysis was performed with the Prism 5.0 software (GraphPad, La Jolla, California). Statistical significance was evaluated by one‐ or two‐way analysis of variance followed by multiple comparisons post‐test as indicated.

## 3 RESULTS

### 3.1 Precise localization of putative vascular wall‐resident MSCs in normal human lung tissue

**FIGURE 1:**
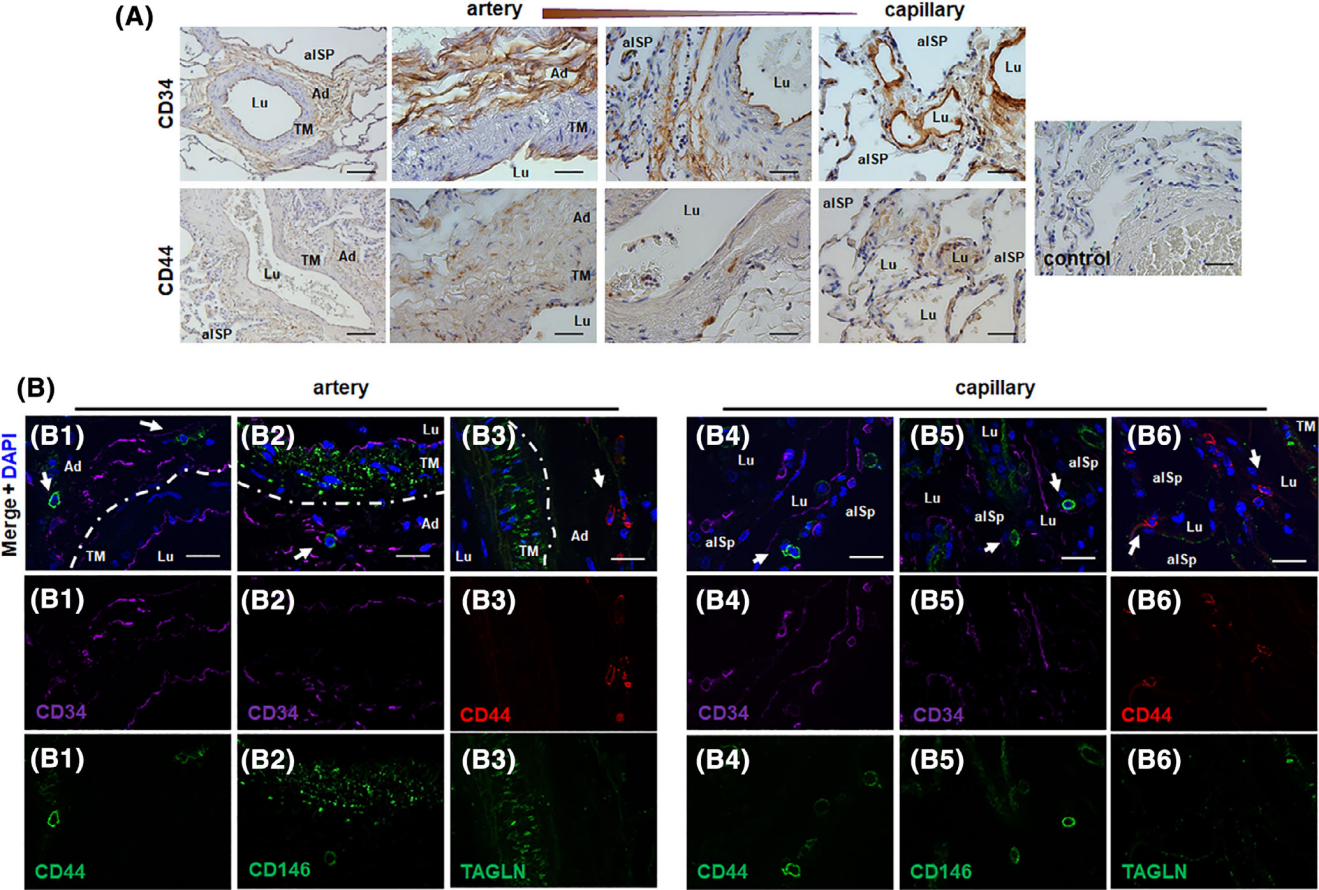
The “vasculogenic zone” of lung blood vessels and co‐localization of mesenchymal stem cell (MSC) marker proteins (putative MSCs). A, Immunohistochemical staining of normal lung tissue sections was performed using antibodies against CD34 (EPC, endothelial progenitor cell marker) and CD44 (MSC marker) and DAB staining (brown). Representative lung photographs of larger blood vessels (with a thick muscular wall, >500 μm external diameter), in intermediate/small‐sized arteries (with thin muscular wall, up to 100 μm in external diameter) and in the microvessels (capillaries) are shown. Nuclei were counterstained with Hemalaun (blue). Lu lumen, TM tunica media, Ad adventitia, alSp alveolar space. Scale bar indicates 100 μm (left panel) and 10 μm (higher magnification images). B, Double‐immunofluorescent staining of normal lung sections was performed using antibodies against the typical MSC maker proteins CD44 (B1, B4) and CD146 (B2, B5) together with the endothelial and hematopoietic progenitor cell marker CD34, and of CD44 together with the smooth muscle cell marker transgelin (TAGLN, B3, B6). The dashes line emphasizes the border between the smooth muscle cell layer (TM) and the adventitia. Nuclei were visualized using DAPI nuclear counterstainings (blue). Representative lung photographs of arteries and microvessels (capillaries) are shown. Scale bar indicates 10 μm

To identify and define putative resident adventitial MSCs in adult human lung tissue, we first performed immunohistologic as well as immunofluorescence staining to characterize these cells in surplus samples of normal lung tissue obtained from donor lungs undergoing thoracic surgery (Figure 1). We used the endothelial and hematopoietic progenitor cell marker CD34 in order to visualize the vasculogenic zone within lung blood vessels (Figure 1A). As expected, in larger (as well as in mid‐sized) lung blood vessels a prominent immunoreactivity of CD34 within the adventitia could be observed, strongly accounting for the presence of the vasculogenic zone. In addition, endothelial cells lining the intima expressed CD34, and the staining seemed to be more intense in the small capillaries. Of note, when using the MSC marker CD44 that was shown to particularly well suited for the detection of VW‐MSCs,^18^, ^24^, ^26^ single CD44‐positive cells could be detected around the lung vessels investigated. These putative MSCs were localized within the adventitia and close to the smooth muscle cell (SMC) layer (tunica media) (Figure 1A and Figure S1A,B). Corroborating these findings, and to characterize their localization pattern within the vessel wall more precisely, we performed double‐immunostainings using marker proteins that distinguish these CD44‐positive MSCs from other vascular cells (Figure 1B and Figure S1C). Again, CD44‐positive cells could be detected within the adventitia around the larger lung vessels investigated; these cells lack endothelial CD34 expression as well as the SMC marker transgelin, a shape‐change sensitive actin cross‐linking/gelling protein involved in calcium interactions and contractile properties of SMC. In addition to the classical MSC marker CD44, the MSC marker CD146 was expressed in putative vascular wall‐resident MSCs in the lungs (LR‐MSCs) (Figure 1B and Figure S1C). Even in the small blood vessels, CD44‐ and CD146‐positive cells can be found in close vicinity to CD34 immunoreactive structures, suggesting the presence of MSCs in the alveolar interstitium.

### 3.2 Isolation and characterization of LR‐MSCs

**FIGURE 2:**
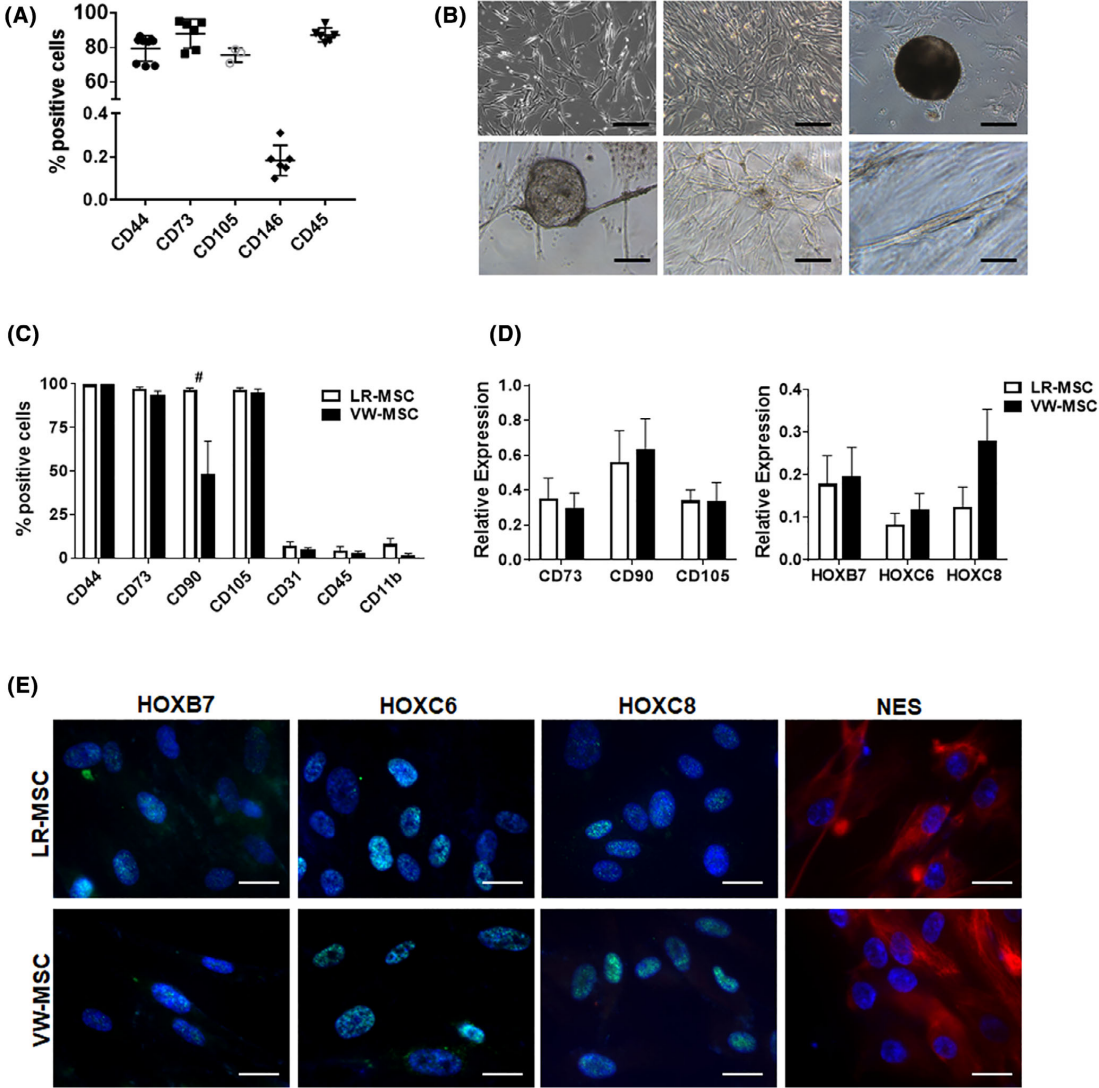
Isolation and characterization of lung‐resident mesenchymal stem cells (LR‐MSCs). A, Normal lung tissue was homogenized by collagenase digestion and the crude cell extract was analyzed by flow cytometry using the indicated (MSC) markers. Data (column scatter plots) include the mean ±SD, n = 4‐7. B, Representative phase contrast micrographs of cells 10 to 12 days after initial plating showed typical mesenchymal (flattened and fibroblast‐like) cell morphology. Cultivated LR‐MSCs form clonally cell aggregates upon prolonged culturing (CFU, colony‐forming units). When LR‐MSCs were embedded in GFR‐Matrigel as 3D‐spheroids, VW‐MSC‐typical in‐gel sprouting and Matrigel invasion (tube formation) was observed. Scale bar 50 μm. C, FACS analysis of cultured LR‐MSCs show that LR‐MSCs are positive for CD90, CD73, CD105, and CD44 but negative for lineage cell markers CD45, CD31, and CD11b indicating no considerable contamination by other types of progenitors. FACS data summarizing for at least four independent experiments (±SEM) are shown. Ex vivo isolated hITA (human internal thoracic artery)‐derived VW‐MSCs were shown as “control.” D, Relative amounts of transcripts of the indicated genes including the VW‐MSC‐specific “HOX code” were further determined by qRT‐PCR in LR‐MSCs and compared to VW‐MSCs (biological replicates: n = 5‐7 per group and gene). Relative transcript levels of analyzed genes were normalized to beta‐actin mRNA (set as 1). E, LR‐MSCs and VW‐MSCs were seeded on gelatin‐coated cover‐slips and HOXB7, HOXC6, HOXC8, and NES expression were detected by immunofluorescence using confocal microscopy (blue, DAPI). Representative photographs from four independent experiments are shown. Scale bar indicates 10 μm

**FIGURE 3:**
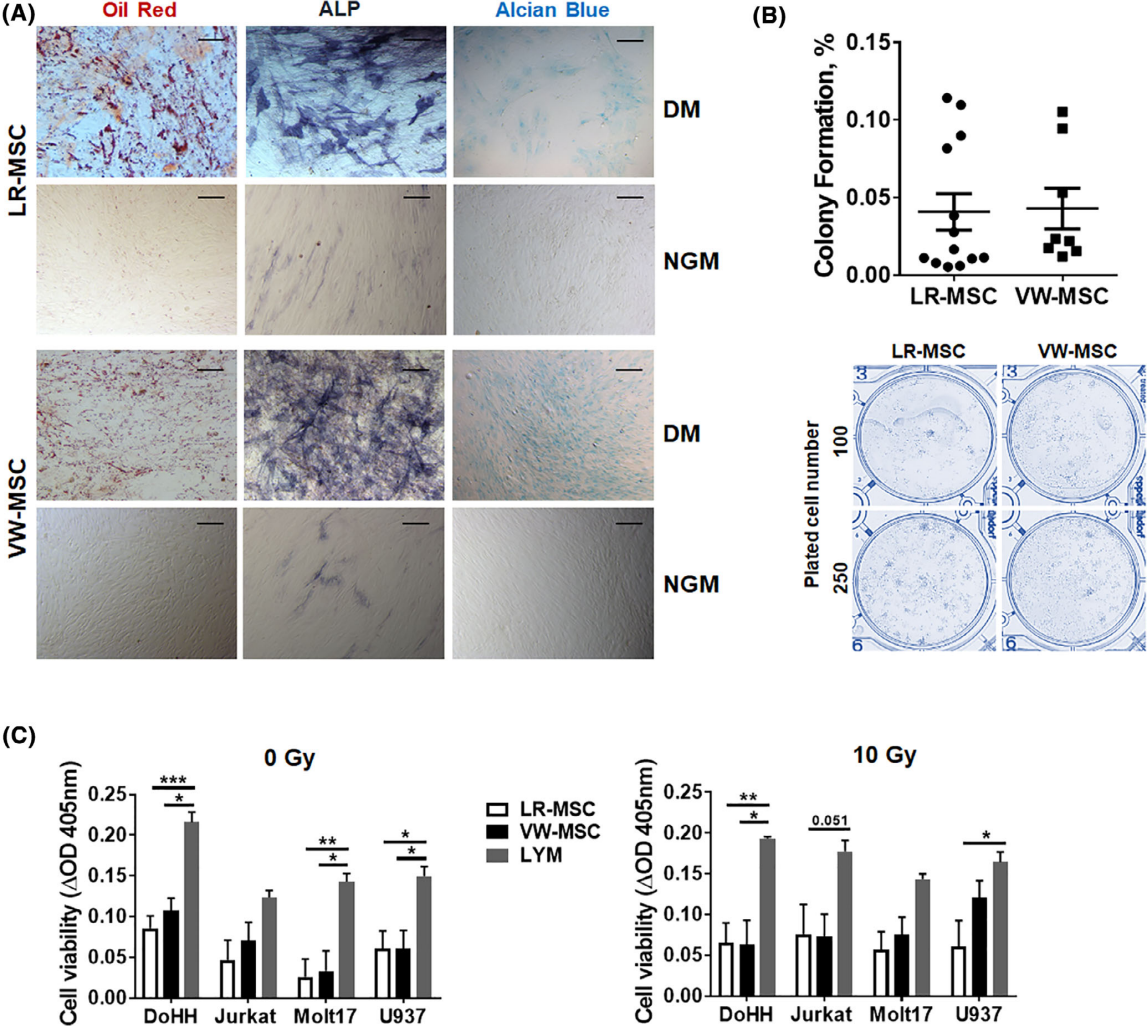
Trilineage differentiation, colony‐forming unit (CFU) and therapeutic potential of LR‐MSCs. A, Trilineage differentiation. Isolated and cultured LR‐MSCs as well as control VW‐MSCs were differentiated into adipocytes, osteocytes and chondrocytes, in vitro. Differentiation was observed within 14 days after induction of differentiation with differentiation media (DM) as shown by Oil red staining for detecting lipid droplets (red) in adipocytes, by histochemical NBT/BCIP staining for detecting alkaline phosphatase activity (ALP, black‐purple) in osteocytes, or Alcian Blue staining (blue) for detecting acidic polysaccharides such as glycosaminoglycans in (eg, the cartilage‐specific proteoglycan aggrecan) in chondrocytes. Representative photographs are shown (biological replicates: n = 3‐4). Magnification ×400. As control, respective cells were cultured in normal growth media (NGM). B, CFU Assay. LR‐MSCs and VW‐MSCs were plated at low densities (100‐1000 cells/well) in plastic culture dishes and subsequently cultured for 10 days Coomassie Brilliant Blue stained colonies were counted and the surviving fraction (colony formation) was calculated. (biological replicates: n = 4‐6 for each group). C, Mixed lymphocyte reaction. Verification of lymphocyte proliferation inhibition: LR‐MSCs as well as control VW‐MSCs were co‐cultured with different lymphoma cells (DoHH2, Jurkat, MOLT17, and U937) for 24 hours. VW‐MSCs were used as positive control. Cell‐cycle arrested, irradiated (10 Gy, 24 hours prior to coculture) MSCs were used to exclude possible effects mediated by their proliferation (right diagram). Cell proliferation was determined using a WST‐1 reagent‐based tetrazolium reduction assay and related to proliferation of lymphoma (LYM) cells alone (biological replicates: n = 4‐5 per group and lymphoma cell line; *P* by two‐way ANOVA followed by post hoc Tukey's multiple comparisons test: *P ≤ .05; **P ≤ .01; ***P ≤ .005; ^#^ *P* ≤ .001)

Next, we attempted to isolate these CD44(+) cells with putative MSC properties from normal human lung tissue (Figures 2 and 3 and Figure S2, S3). Flow cytometry analysis of crude cell extracts using collagenase digestion of ex vivo isolated normal lung tissue revealed, that most of the cells (75%‐90%) expressed the classical MSC markers CD44, CD73, and CD105 (Figure 2A). Surprisingly, CD45 was also detected in such high amounts. We attribute this to the fact that the lung as compared to small human internal thoracic artery (hITA,) vessel fragments were “filled” with infiltrated immune cells. In addition, the isolation protocol for vascular wall‐resident MSCs (VW‐MSCs), initially designed to isolate VW‐MSCs from ex vivo isolated small hITA fragments^18^, ^24^, ^26^ liberates loosely integrated adventitial MSCs, and here in the lungs infiltrated (CD44‐positive) immune cells efficiently, whereas tighter lung structures remained unaffected. Of note, a small number of cells namely 0.183 ± 0.029% of isolated cells expressed the MSC marker CD146 (Figure 2A). In order to enrich the cells efficiently and get rid of contaminating CD45‐positive cells, we further used plastic adherence and medium selection.^24^, ^25^, ^28^ Therefore, lung cell homogenates were cultured on plastic dishes in MSC‐specific medium, and free‐floating cells were washed away (initially 4‐6 hours after the first attachment of cells, and again after 24 hours of culturing). Cultured LR‐MSCs showed flattened MSC‐typical morphology (Figure 2B). When LR‐MSCs were embedded in GFR‐Matrigel as 3D‐spheroids, VW‐MSC‐typical in‐gel sprouting and Matrigel invasion (tube formation) was observed. Flow cytometry analysis of cultured LR‐MSCs then revealed that the purity of these cell preparations was routinely >90%‐95% as analyzed by expression of the MSC marker panel including CD105, CD73, CD44, and CD90. The absence of contaminating cell types such as mature EC or EPCs, and hematopoietic cells was demonstrated by lack of CD31, CD45, and CD11b expressing cells (Figure 2C and Figure S2). We included here and for the following analyses VW‐MSCs isolated from hITA as a “control.” According to our hypothesis, we suggest that LR‐MSCs could be considered as VW‐MSCs. To strengthen our suggestion, we investigated the expression of the VW‐MSC‐specific HOX code in LR‐MSCs (Figure 2D,E). Similar *HOXB7*, *HOXC6*, and *HOXC8* mRNA expression levels as analyzed by quantitative real‐time RT‐PCR were determined for both MSCs (Figure 2D). As visualized by immunofluorescence, increased cytoplasmic as well as a prominent nuclear localization of the HOX proteins was observed in LR‐MSCs and hITA‐derived VW‐MSCs (Figure 2E). The propensity of isolated LR‐MSCs to differentiate toward adipocytes, osteoblasts, and chondrocytes, was tested by plating and culturing the cells in appropriate differentiation media for additional 14 days (Figure 3). Adipogenic, osteogenic as well as chondrogenic differentiation of LR‐MSCs was comparable to those of VW‐MSC (Figure 3A). In addition, the propensity for CFU formation was comparable in both MSCs (Figure 3B). To confirm that LR‐MSCs, similar like VW‐MSCs, were able to contribute to the morphogenesis of functional blood vessels, in vivo,^18^, ^25^ both MSC isolates were subcutaneously transplanted together with endothelial cells (as angiogenic stimulus) in Matrigel into immune‐deficient NMRI mice (Figure S3). After 14 days, plugs were re‐isolated. Functionally perfused blood vessels within the plugs are identified by presence of erythrocytes (red cells) as detected by phase contrast microscopy (Figure S3A, left panel). Formation of new blood vessels derived from implanted cells within the plugs was further demonstrated by the presence of vessels lined by (human) CD31‐positive endothelial cells while being mouse CD34‐negative, which were stabilized by transgelin (TAGLN)‐reactive mural cells that displayed a flattened and elongated phenotype, indicating the potential differentiation of co‐implanted LR‐ and VW‐MSCs toward vascular mural cells (Figure S3A, right panel). The potential of MSCs isolated from normal lungs to modulate immune cells was investigated by testing their ability to inhibit lymphocyte proliferation using an allogeneic mixed lymphocyte reaction with different human nonadherent lymphoma cells as mitogens. Cell‐cycle‐arrested, irradiated (10 Gy) LR‐MSCs and control VW‐MSCs were used to determine background proliferation during the measurements. LR‐ and VW‐MSCs significantly suppressed the proliferation of the different lymphoma cells as well as of peripheral blood mononuclear cell (PBMC) derived from healthy donors in a similar manner (Figure 3C and Figure S3B,C).

Conclusively, LR‐MSCs reside within the lung tissue predominately with the vascular stem cell niche. Together with the highly similar MSC characteristics of VW‐MSCs, including the “VW‐MSC‐specific HOX code,” LR‐MSC were practically indistinguishable from VW‐MSCs. Thus, LR‐MSC should be considered as VW‐MSCs.

### 3.3 LR‐MSC and the “vasculogenic zone” of lung blood vessels in lung cancer tissues

**FIGURE 4:**
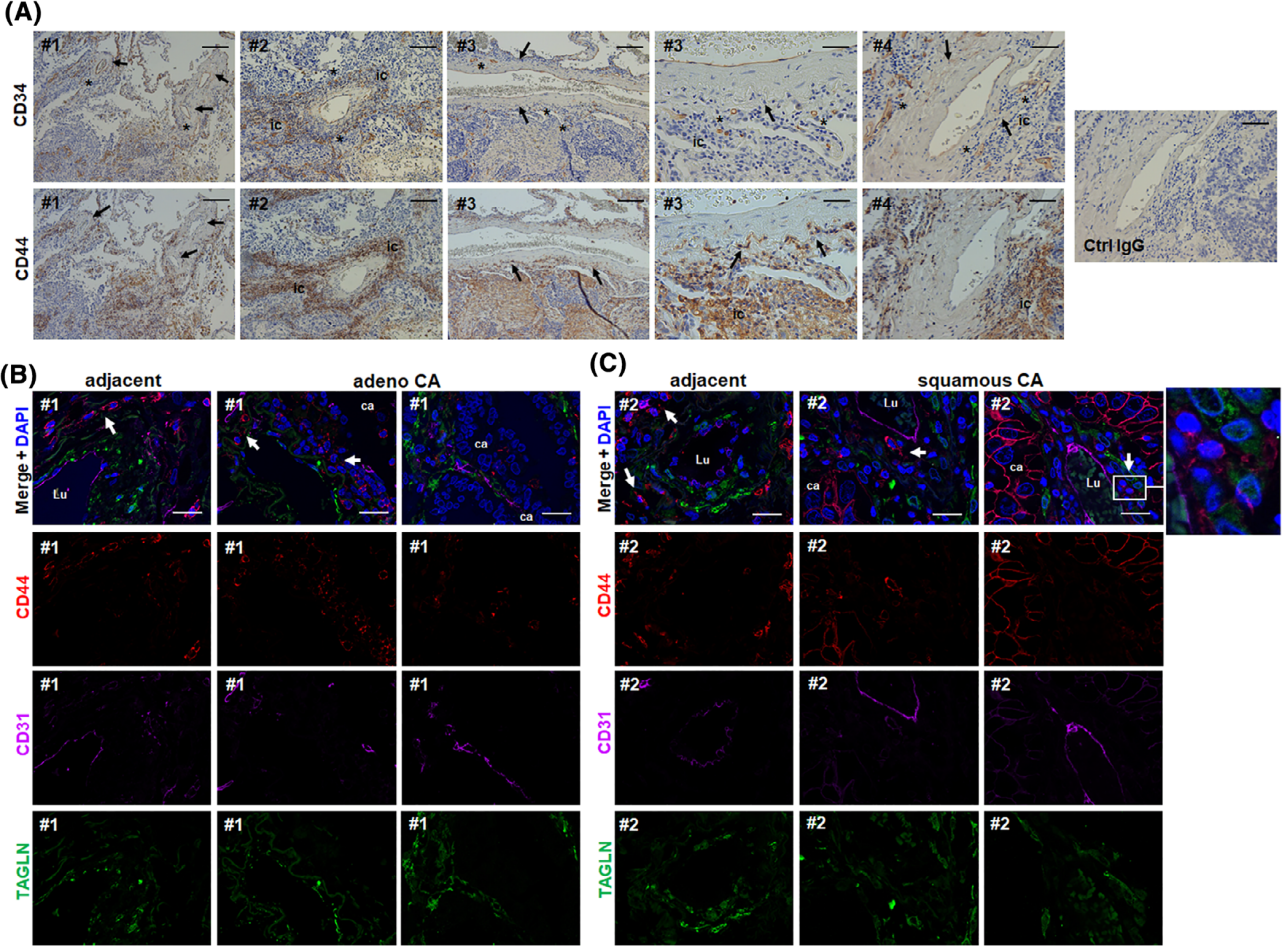
The “vasculogenic zone” of lung blood vessels in NSCLC tissues and co‐localization of CD44‐positive vascular stem cells (LR‐MSCs). A, Immunohistochemical staining of paraffin‐embedded human NSCLC tissue sections were performed using antibodies against CD34 and CD44 in combination with DAB staining (brown). Representative photographs of large and intermediate‐sized lung blood vessels are shown. Arrows point toward cells residing in the vasculogenic zone. Asterisks highlight the vanishing of CD34 immunoreactive cells indicating that endothelial progenitor cells mobilize out of that zone (toward the tumor). Nuclei were counterstained with Hemalaun (blue). # numbers indicate NSLC tissues from different patients. ic, infiltrating immune cells. Scale bar indicates 100 μm (left panels) and 20 μm for the higher magnification images (right panel). B and C, Triple‐immunofluorescent staining of NSCLC specimen were performed using antibodies against the typical MSC maker protein CD44 (red) together with the smooth muscle cell marker transgelin (TAGLN, green) and the EPC marker CD34 (purple). Nuclei were visualized using DAPI (blue). Representative lung photographs of larger tumor blood vessels as found in adenocarcinoma (adeno‐CA) (B) and squamous cell carcinoma (squamous CA) (C) as well as of arteries present in adjacent normal lung tissue are shown. Different regions (blood vessels) of the same donor (#) are presented. Arrows point toward CD44‐positive cells residing in the vasculogenic zone. Lu, vessel lumen; ca, carcinoma cells. Scale bar indicates 10 μm

For now, it is clear that MSCs reside also in the lungs where they are important regulators of tissue homeostasis and harbor the capacity to suppress inflammation and promote repair. However, lung MSCs can be even affected by pathological trigger and/or upon pathogenesis and then contribute to the disease. Therefore, we analyzed the expression of vascular stem cell markers within human NSCLC tissue (Figure 4). CD34, as similarly shown for normal lung tissue, marked the adventitial stem cell zone; CD44‐positive cells can even be found here (Figure 4A). As CD44 seems to be highly expressed on immune cells, areas of infiltrating CD44(+) immune cells (ic) can be detected. Of note, whereas CD34 EPCs seems to become reduced in the vasculogenic zone (Figure 4A, asterisks), potentially migrating toward areas/clusters of tumors cells, CD44 immunoreactivity seemed to increase in numbers in the vasculogenic zone (Figure 4A, arrows). In order to specify these findings, and to characterize MSC/EPC localization pattern within and close to the vessel wall in more detail, we performed triple‐immunostainings of adenocarcinoma and squamous cell carcinoma tissue sections (Figure 4B,C and Figure S4). In larger vessels of tumor adjacent lung tissue as well as in normal/healthy lung tissue, CD34 clearly marks the vasculogenic zone, where also CD44‐positive LR‐MSCs reside, which coexpress the MSC marker CD146 (Figure S4A,B). In blood vessels close by and within NSCLC areas CD34(+) EPCs were found less frequently within the vessel wall but more in tumor region, strongly confirming their role in tumor vascularization. CD44(+) and CD146(+) cells, however, seemed to be increased in numbers within the vascular wall and in close vicinity of vascular structures (as well as within NSCLC tissues) (Figure 4B,C and Figure S4). Although highly desirable and potentially unraveling lineage tracing could not be performed at that stage, these results strongly account for an involvement of LR‐MSCs in the progression of NSCLC.

### 3.4 The prognostic value of CD44 and of “HOX code” expressions

**FIGURE 5:**
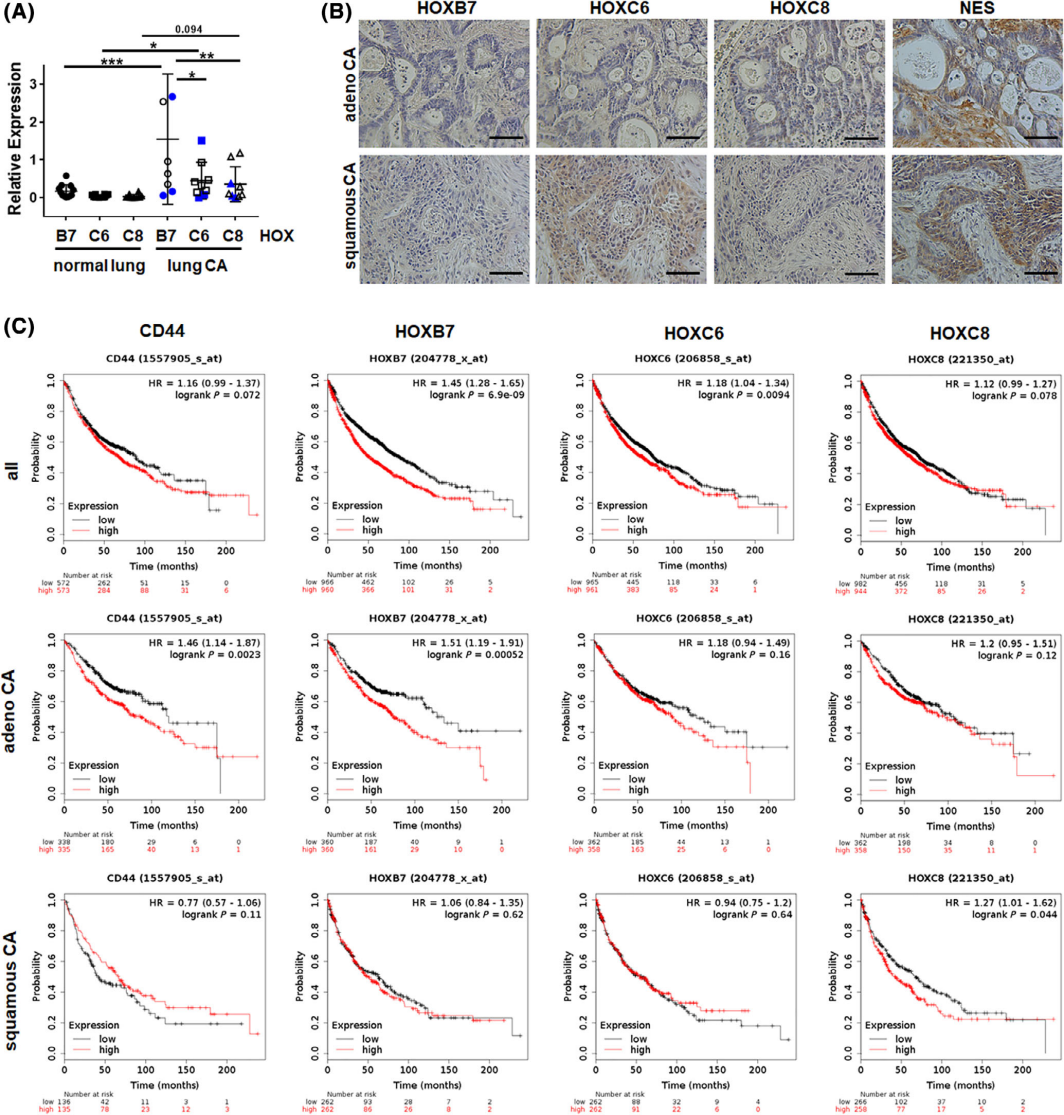
Expression levels of the VW‐MSC‐specific “HOX code” in human NCSLC tissues and the prognostic impact. A, HOXB7, HOXC6, and HOXC8 mRNA expression levels were analyzed in total RNA isolates generated from normal lung or lung carcinoma (CA) tissue using qRT‐PCR. Data are presented as mean ± SEM (normal lung tissue: n = 12 per gene; NSCLC/lung CA: n = 9 per gene). Blue symbols depict squamous CA values. *P*‐values were indicated: *P ≤ .05, **P < .01, ***P ≤ .005 by one‐way ANOVA followed by post hoc Tukey's multiple comparisons test. B, Paraffin‐sections of human NSCLC specimen were stained for the indicated HOX antibodies in combination with DAB. Sections were counterstained using hematoxylin. Representative images are shown. Magnification ×20 (phase contrast), scale bar: 40 μm. C, Survival curves were plotted for all NSCLC patients (CD44: n = 1145; HOX: n = 1926) (left panel), adenocarcinoma (adenoCA) patients (CD44: n = 673; HOX: n = 720) (middle panel), and squamous cell carcinoma (squamous CA) patients (CD44: n = 271; HOX: n = 524) (right panel). Data was analyzed using Kaplan‐Meier plotter for lung cancer (www.kmplot.com). Expressions in cancer tissues above the median are indicated in red line, and expressions below the median in are summarized in black line. HR, hazard ratio

As aberrant HOX gene expression is already found in several cancers including NSCLC, functioning either as oncogenes sustaining cell proliferation or as anti‐oncogenes (tumor‐suppressive) by controlling cell differentiation, we investigated HOX expression in normal lung tissue as well as in NSCLC tissue (Figure 5). Quantitative real‐time RT‐PCR (qRT‐PCR) analyses of total RNA isolates generated from directly ex vivo isolated normal lung or lung carcinoma tissues revealed a significant upregulation of *HOXB7* and *HOXC6*, as well as *HOXC8* mRNA by tendency expression in the NSCLC tissues (Figure 5A). Immunohistochemistry analysis of respective NSCLC tissue further confirmed increased HOXC6 and HOXC8 immunoreactivity with nuclear as well as partially cytoplasmic staining patterns in most of the tissues investigated (Figure 5B and Figure S5A). Unfortunately, we could not detect HOXB7 by this method, which is most likely due to the imperfection of the HOXB7 antibody in combination with paraffin‐embedded tissue sections. Quantification of HOX‐expressing cells within normal lung and NSCLC tissue confirmed an increase of cells expressing the LR‐/VW‐MSC‐specific HOX code in NSCLC tissue, as well as the increase of putative LR‐MSCs coexpressing the classical MSC marker CD146 and the stage‐specific embryonic antigen‐4 (SSEA4) as additional MSC marker (Figure S5B). With respect to the hypothesis that lung cancers might be originated from resident stem cells, and the most originating sites of different types of lung cancer correlated with distinct airway stem cell niches, we conclude here that an abnormal expression of HOX genes and in particular of our VW‐MSC‐specific HOX genes in NSCLC could be involved in the transformation of adult stem cells into cancer (stem) cells; and/or at least contribute to important NCSLC histological tumor type features. We then studied the relationship between mRNA expressions of our VW‐MSC/LR‐MSC markers (CD44 and the “HOX code”) and the clinical outcome using a Kaplan‐Meier plotter (Figure 5C). Survival curves were plotted for all NSCLC patients and separately for adeno‐ and squamous NSCLC patients. CD44 high mRNA expression was found to be correlated to worse overall survival (OS) for all NSCLC patients by tendency, but significantly when considering adenoCA patients only. CD44 expression in squamous CA did not significantly affect OS. High HOXB7 mRNA expression levels showed a similar trend. HOXB7 high mRNA expression correlated to a significantly worse OS for all NSCLC patients and when considering adenocarcinoma patients only, whereas squamous NSCLC patients were not affected (Figure 5C). High HOXC6 as well as HOXC8 mRNA expression correlated with a significantly worse OS for all NSCLC patients, whereas the effect was less prominent in the respective histology subgroups (Figure 5C).

Thus, an increase in VW‐MSC‐specific “HOX code” expression levels, namely *HOXB7*, *HOXC6, and HOXC8* mRNA expression levels contribute to a less favorable prognosis in NSCLC patients, although the exact prognostic significance of these genes needs to be further elucidated.

### 3.5 Potential interactions of LR‐MSCs and lung cancer cells

**FIGURE 6:**
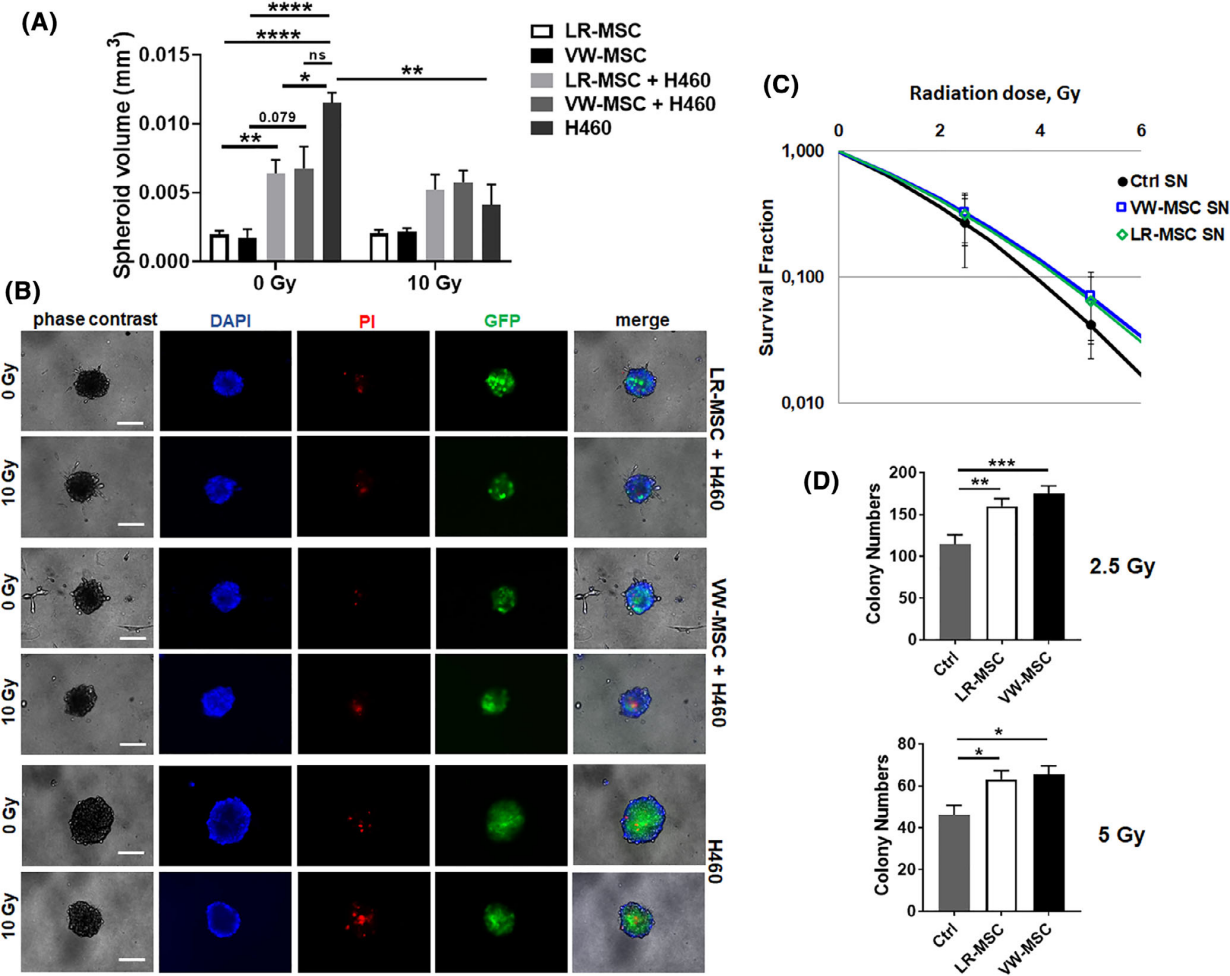
Interaction of LR‐ and VW‐MSCs with lung cancer cells. A, NCI‐H460 lung cancer cells labeled with green‐fluorescent protein (GFP) were cultured alone or co‐cultured with LR‐MSCs or VW‐MSCs in hanging drops for 24 hours. After formation of spheroids, cells were plated in GFR‐Matrigel mixed with normal growth medium (1:2, vol/vol) and left untreated or irradiated at 10 Gy. Spheroids growth were measured after additional 48 hours of cultivation and respective volumes were calculated. Graphs depict the measurements from at least three independent experiments (H460, n = 3, LR‐MSC and LR‐MSC + H460, n = 6, VW‐MSC and VW‐MSC + H460, n = 5) where at least 10 spheroids per condition each were measured. *P < .05, **P < .01, ****P < .001 by one‐way ANOVA followed by post hoc Dunnett's test. B, Cell death was analyzed afterwards (48 hours' time point) by fluorescence microscopy using propidium iodide. DAPI was used for nuclei staining. Representative phase contrast images and simultaneously recorded fluorescent photographs from three individual experiments are shown (48 hours' time point). Scale bar represents 25 μm. C, Lung cancer cells were plated at low densities (CFU assay), irradiated with indicated doses (0, 2.5, 5 Gy) and further incubated for additional 10 days in conditioned media (SN) derived from cultured LR‐ and VW‐MSCs. Quantification of grown/surviving colonies was performed after Coomassie Brilliant Blue staining (Ctrl SN, n = 3, LR‐MSC SN, n = 6, VW‐MSC SN, n = 5). *P* by two‐way ANOVA, followed by post hoc Dunnett's multiple comparisons test: *P ≤ .05, **P ≤ .01, ***P ≤ .005

We then investigated if and how LR‐MSCs would affect adjacent lung cancer cells. Therefore, LR‐ and VW‐MSCs were directly co‐cultured with H460 lung cancer cells using spheroids embedded in GFR‐Matrigel (3D co‐culture) (Figure 6). LR‐MSCs significantly reduced the growth of H460 cells upon direct co‐culture, similarly to the action of VW‐MSCs (Figure 6A). Upon radiation treatment, as additional stressor, the radiation‐induced growth delay of H460 cells was limited when co‐cultured with MSCs. When lung cancer cells were cultured alone, the reduction of spheroid growth upon radiation treatment was accompanied by the presence of tumor cells that were permeable for propidium iodide, and thus showed radiation‐induced cell death (Figure 6B). In contrast, the reduced growth of lung cancer cell‐MSC co‐cultures and the reduced growth delay of spheroids upon radiation treatment were not accompanied by increased cell death levels, indicating that LR‐ and VW‐MSCs were able to directly affect lung cancer cells proliferation, and potentially mediate a more resistant phenotype (Figure 6B). Conformingly, MSC‐derived factors (by using conditioned media from LR‐ and VW‐MSCs cultures) were able to limit the reduced clonogenic survival of H460 cells upon cellular stress induced by radiotherapy (Figure 6C,D).

Conclusively, whereas in the healthy situation LR‐MSCs may function to regulate pulmonary tissue repair and/or regeneration, lung MSCs can be affected upon pathogenesis and then contribute to the disease, potentially as mesenchymal cells of the tumor stroma that impacts on lung cancer progression (and therapy resistance) as shown here.

## 4 DISCUSSION

**FIGURE 7:**
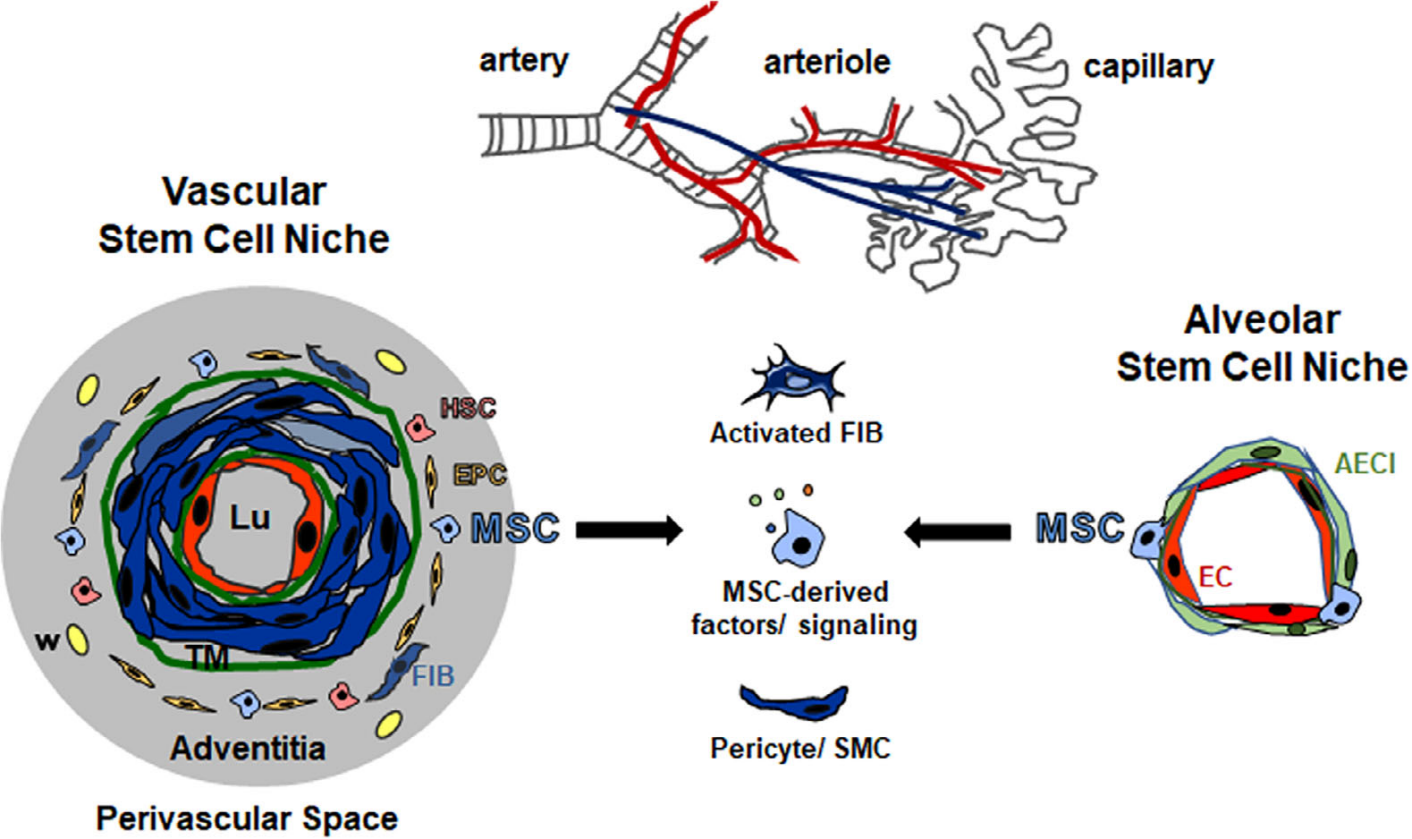
Lung‐resident MSCs: Localization and potential contributions to lung cancer. Vascular wall‐resident mesenchymal stem cells (VW‐MSCs; blue) are localized in the prominent adventitial stem cells niche, the so‐called vasculogenic zone of large and mid‐sized arteries (left side). The vasculogenic zone is a vascular mural zone located within the adventitia and close to the tunica media (TM). The border between media and adventitia is marked by outer elastic membrane (green). This vascular stem cell niche further harbors endothelial progenitor cells (EPC) and hematopoietic stem cells (HSC). Within lung tissue, VW‐MSCs could most likely be designated as LR‐MSCs, as LR‐MSCs were not only lung‐resident but also tissue‐specific and in particular showed tissue specificity of the vessel wall. The alveolar stem cell niche (right side) combines the vascular niche with the airway niche. Within the smallest blood vessels (capillaries) LR‐MSCs can be found in the small alveolar interstitium, close to the capillary endothelial cells that share their intermediate basement membrane with sheet‐like alveolar epithelial cells (type I, AECI). LR‐MSCs contribute to the maintenance of tissue integrity in adult lungs. Following a pathological trigger, however, lung injury might at least be partially mediated by endogenous MSCs. Due to their vascular localization, lung‐resident MSCs could be the first line cells, which are available on the basis of their anatomic location as first point of contact for tumor cells and secreted factors. Mobilized and/or activated MSCs bear the ability to migrate toward primary tumors and metastatic sites, thereby impacting on lung cancer progression, either by an altered tumor‐promoting secretory profile, by differentiating into pericytes and smooth muscle cells (SMCs) and/or into activated fibroblasts (myofibroblasts and cancer‐associated fibroblasts). Moreover, upon lung damage, tissue‐resident MSCs could be one cell of origin for cancer development or progression. Finally, in terms of manufacturing exogenous MSCs with superior repair capabilities, lung MSCs itself might be logically more efficient for a therapeutically approach than MSCs derived from other tissues for the treatment of lung diseases

LR‐MSCs were already successfully isolated from healthy and diseased human lung specimen. Although described as located perivascular, the cellular identity of primary lung MSCs remained elusive. Here we investigated the vascular nature of LR‐MSCs. Within normal human lung and NSCLC tissue, LR‐MSCs predominately reside within the vascular stem cell niche, namely the vasculogenic zone of adult lung arteries, a vascular mural zone located within the adventitia (Figure 7). The distribution of MSC‐like cells throughout the whole body, or more precisely cells that express natively, before culture, commonly used MSC markers was already suggested, as these cells were found to be localized perivascular throughout fetal and adult human organs.^32^ Although the refined analysis strongly indicated an MSCs nature, it was suggested later on that all adult perivascular MSC (‐like) cells are pericytes.^33^, ^34^ Pericytes (initially identified as “Rouget cells” in 1873) per se are contractile mural cells surrounding the endothelial cells of blood microvessels. Although pericytes might be characterized by a certain cellular plasticity, the commonly applied defining criterion is their presence in microvessels (capillaries, postcapillary venules, and/or terminal arterioles).^35^, ^36^ Electron microscopy is classically used to define mature pericytes being embedded within the vascular basement membrane in smallest blood vessels.^37^ Thus, based on their anatomic location it is clear that pericytes are not located in the adventitia and/or perivascular and are not adventitial (VW‐) MSCs. For the correct understanding of vascular wall biology and further for potentially regenerative approaches it is important to use a consistent—anatomically correct—nomenclature.^17^, ^20^ The vascular adventitia within adult blood vessels represents the interface between vessel wall and surrounding tissue, namely the perivascular space(s) that vary in dimension according to the type of blood vessel.^38^, ^39^ Herein, the vascular stem cell niche, a considerable reservoir for different types of progenitor and stem cells, including MSCs, with the potential to differentiate into vascular and nonvascular cell types (Figure 7) is located.^17^, ^20^, ^22^, ^40^, ^41^, ^42^

We show here that LR‐MSCs were not only tissue‐resident as revealed by their localization within the adventitia, but also tissue‐specific and in particular showed tissue specificity of the vessel wall. Primary LR‐MSCs showed the typical MSC characteristics in vitro. Plastic adherence, flow cytometric analyses of the classical phenotypic MSC profile, colony‐forming capacities, and the differentiation potential among the mesodermal lineages including vascular mural cells were similar to those properties of ex vivo isolated adventitial VW‐MSCs obtained from hITA fragments. With respect to their therapeutic potential, LR‐MSCs suppressed lymphocyte proliferation in a similar manner as VW‐MSCs. Most importantly, LR‐MSCs expressed the VW‐MSC‐specific gene code, the “HOX code,” a characteristic to discriminate VW‐MSCs from phenotypical similar cells. HOX genes comprise a family of (evolutionary conserved) regulatory genes that encode for transcription factors controlling the activity of other functionally related genes as part of morphogenesis. HOX genes regulate the structure the embryo along the body's longitudinal axis.^43^, ^44^, ^45^ The characteristic embryonic HOX gene expression profiles, the so‐called “HOX code,” is sustained in adults, where HOX genes regulate cell specification and tissue differentiation according to the body region.^46^ Accordingly, the topographical specificity of HOX genes is maintained in tissue‐typical MSCs and further on after differentiation, which indicates that HOX genes are decisive for the specification of MSC identity. As HOX gene expression profiles mirrors that of the developmental origin of MSCs, HOX gene expression can be used to distinguish different stem cell populations and in particular MSCs of functionally distinct tissues.^24^, ^46^, ^47^ VW‐MSCs isolated from the vascular wall of adult blood vessels, express particularly the HOX genes HOXB7, HOXC6, and HOXC8.^24^ This HOX code can be even used to distinguish these vascular wall‐resident MSCs from phenotypically similar cells, like other vascular cells and/or fibroblasts.^24^, ^25^, ^28^ Based on the similar expression patterns of the VW‐MSC‐specific HOX code reported here for LR‐MSCs, together with the highly similar LR‐MSC characteristics as compared to VW‐MSCs, we suggest that LR‐MSC should be considered as VW‐MSCs. Tissue‐resident lung‐derived MSC were already shown to share many similarities with the well‐characterized MSCs classically isolated from bone marrow, but these similarities were predominately of phenotypic nature. More interestingly, LR‐MSC showed a number of lung‐specific properties. Whereas the colony‐forming capacity seemed to be similar in both MSCs derived from different organs, LR‐MSC for example secreted significantly higher amounts of the chemokine monocyte chemotactic protein‐1 as compared to bone marrow (BM)‐MSCs.^48^ In particular, typical lung‐related genes such as FOXF1 and HOXB5 were found to be expressed higher by lung‐derived MSC. This again strengthens our previous findings. Beside the VW‐MSC‐specific HOX code members HOXB7, HOXC6, and HOXC8, which showed the highest impact of differential expression in VW‐MSCs, HOXB5 was already reported to be VW‐MSC‐typical.^24^ This again highlights the similarity between LR‐MSC and VW‐MSCs.

NSCLC accounts for the majority of lung malignances.^49^ Three predominant histological subtypes can be distinguished: squamous cells carcinoma (SCC), adenocarcinoma, and large cell carcinoma. With regard to the findings that a distinct phenotype of lung cancer, shares characteristics with the corresponding regional (epithelial) stem cell population and that different types of lung cancer are additionally correlated with distinct airway stem cell niches, it is supposed that lung tumors may originate from local stem cells that reside within these distinct airway stem cell niches.^50^, ^51^ Upon oncogenic stimuli, the activation of proliferation and survival pathways then turn the finely regulated growth potential of normal (stem) cells into uncontrolled growth of the respectively transformed cancer cells.^52^, ^53^, ^54^ For example, basal cells of proximal airway were associated with SCC, bronchiolar exocrine club cells (formerly known as Clara cells) as well as pulmonary neuroendocrine cells were associated with small cell lung carcinoma, and stem cells from the bronchoalveolar duct junction region were associated with adenocarcinoma and bronchoalveolar carcinoma.^50^, ^55^ Moreover, the stem cell niche usually dictates stem cell function. Thus, tissue‐resident stem cells and their respective niches as stem cell specific microenvironments were considered as key players in the initiation, malignance, and resistance of lung cancer.

Accordingly, whereas in the normal healthy situation the plasticity of lung MSC may be essential for lung homeostasis, errors in the control could lead to de‐differentiation of stem cells thereby causing cancer or at least differentiate to heterogeneous and nontumorigenic cells that ultimately define the histological type of the cancer. Conformingly, stem cell‐related marker expressions were correlated with histologic subtypes. CD44 expression for example was associated with low pathologic stage in lung adenocarcinoma, while expression of the stem cell transcription factor Nanog correlated with high histologic grade that was associated with a poor prognosis.^56^ CD44 was found to be expressed in peri‐bronchial mucus glands and basal cells of the bronchial epithelium, but not in completely differentiated epithelial cells.^56^ Classical MSC markers (CD90, CD44, CD105, CD73) were also known to be expressed on cancer (stem) cells that in turn are responsible for conferring the ability of tumor regeneration. Although a certain tumor phenotype may predominately be characterized by the genetic alterations prevailing in the majority of respective cancer cells found, evidence is accumulating that cancers of distinct subtypes within an organ may derive from different “cells of origin.”^57^

As generally known from the rather low numbers of tissue‐resident MSCs in normal tissues,^2^, ^58^, ^59^ LR‐MSCs were found to be increased in numbers in NSCLC tissues. Together with the elevated MSC marker levels and particularly elevated HOX levels in NSCLC lung tumors reported here, NSCLC may at least partially derive from LR‐MSCs and/or contribute to important and decisive (stromal) features characterizing the histologic subtypes. Concerning the first assumption, most of the HOX genes were found silent or expressed at low levels in normal lung, while the great majority of HOX genes were expressed in lung cancer at high levels.^60^ Of note, it was demonstrated that alteration in HOX gene expression in lung cancer mainly concerns the HOX B and C loci.^61^ Increased expression of HOXB7 (within a 10‐gene prognostic signature) was shown to correlate with poor prognosis in lung adenocarcinoma.^62^ HOXB7‐overexpressing tumors were shown to be enriched in gene signatures characterizing adult stem cells as well as pluripotent stem cells. As a direct target of HOXB7 emerging cancer gene and pluripotency factor LIN28B was identified that sustained the expansion of a subpopulation of cells with stem cell characteristics.^62^ Likewise, the oncogenic potential of HOXC6 was investigated. HOXC6 expression was significantly increased in the majority of NSCLC tumor samples in as compared to healthy control lungs, which suggested HOXC6 as a novel biomarker for the diagnosis and treatment of NSCLC.^63^ Accordingly, ectopic expression of HOXC6 in NSCLC cell lines promoted proliferation, migration, and invasion of respective NSCLC cells.^63^ From the three MSC‐specific HOX candidates, HOXC8 was also found to be upregulated in clinical NSCLC specimens, and the high expression of HOXC8 correlated with tumor grade, tumor node metastasis stage, and poor relapse‐free survival for lung cancer patients.^64^ Mechanistically, HOXC8 functioned as a transcriptional activator of transforming growth‐factor beta signaling pathway, finally mediating increased proliferation, anchorage‐independent growth and migration of NSCLC, as well as chemoresistance and antiapoptosis in NSCLC.^64^ In line with those findings, and according to the second assumption that LR‐MSCs could contribute as heterogeneous and nontumorigenic cells to lung cancer, which ultimately define the histological type of NSCLC, we report here that LR‐MSCs and VW‐MSCs impacted on NSCLC proliferation and survival.

In the normal healthy situation, the lung as “tumor microenvironment” and particularly LR‐MSC usually suppresses malignant transformation. MSCs including LR‐MSCs isolated from healthy tissues can limit the proliferation of solid tumor and leukemia cell lines as well as tumor cell growth dose‐dependently.^65^, ^66^, ^67^ MSC trapping to lung tumors after systemic infusion was even used for lung cancer‐targeted drug delivery by using MSCs as vehicles that were loaded with anti‐cancer drugs (docetaxel) containing nanoparticles.^68^ In contrast to the “protective” action of circulating MSCs once therapeutically applied that is attributed to soluble factors secreted by these cells, LR‐MSCs in their native environment turned out to orchestrate the fate of tumor cells by mediating either tumorigenic or antitumor activity.^65^ Due to their vascular localization, LR‐MSCs could be the first line cells, which are available on the basis of their anatomic location as first point of contact for tumor cells and secreted factors (Figure 7).^17^, ^24^, ^41^ Mobilized and/or activated MSCs bear the ability to migrate toward primary tumors and metastatic sites, thereby impacting on lung cancer progression. Tumor‐infiltrating MSCs then evolve as tumor‐associated MSCs and/or myofibroblasts and cancer‐associated fibroblasts that critically affect the tumors survival, proliferation, migration and invasion, as well as the immune status of the tumor microenvironment.^69^ LR‐MSCs were already shown to affect the NSCLC phenotype. Conditioned media derived from LR‐MSCs either isolated from normal lung tissue or from NSCLC adjacent (pathological) tissues promoted NSCLC cells' proliferation, migration, autophagy and epithelial to mesenchymal transition, whereby the pathological MSCs exhibited a more profound effect.^70^ This NSCLC stimulation was mediated through niche‐specific MSC microvesicles.^71^ Therefore, different targeting strategies would be required in accordance with niche/disease stage to inhibit lung cancer progression by potentially modulating the interaction between LR‐MSCs and NSCLC.^70^, ^71^

## 5 CONCLUSION

LR‐MSCs predominately reside within the vasculogenic zone of adult lung arteries. Together with the highly similar MSC characteristics of VW‐MSCs, LR‐MSC should be considered as VW‐MSCs. LR‐MSCs contribute to normal lung homeostasis by their capacity to suppress inflammation and their potential to differentiate into several cell types, thereby promoting lung repair. A detailed understanding of the conserved biology in particular of LR‐MSCs and their respective niches will gain mechanistic knowledge of the behavior of these cells during organ homeostasis and tissue repair, and foster strategies, to manipulate these cells on site (eg, protecting the endogenous stem cell pool) and/or isolate respective cultures with superior repair capabilities. However, following lung damage, LR‐MSC seem not to appear sufficient for tissue protection or repair. LR‐MSCs contribute to lung diseases like fibrosis or particularly lung cancer as investigated here. The MSC‐specific HOX code turned out to be highly expressed in NSCLC. LR‐MSC activities for the NSCLC cells their self (as a potential cellular origin) and/or as mesenchymal cells of the tumor stroma may critically affect lung cancer progression. Comprehensive evaluation of expression and prognosis of HOX genes will be a benefit for the better understanding of heterogeneity and complexity in the molecular biology of NSCLC, paving a way for more accurate prediction of prognosis and discovery of potential drug targets for NSCLC patients.

## CONFLICT OF INTEREST

T.H. declared advisory role for Bristol Myers Squibb and speakers' bureaus for Bristol Myers Squibb, Merck Sharp & Dohme, Chugai Pharma; however, no commercial interest in correlation with this manuscript. The other authors declared no potential conflicts of interest.

## AUTHOR CONTRIBUTIONS

L.K., C.H.: collection and/or assembly of data, data analysis and interpretation, revision, final approval of manuscript; A.S., T.H., C.A.: collection and/or assembly of data, provision of study material or patients, revision, final approval of manuscript; V.J.: administrative support, financial support, final approval of manuscript; D.K.: conception and design, collection and/or assembly of data, data analysis and interpretation, manuscript writing, financial support, revision, final approval of manuscript.

## Supporting information


**Data S1.** Supporting Information.

## Data Availability

The data and code that support the findings of this study are available from the corresponding authors upon request.

## References

[1] Galipeau J , Sensebe L . Mesenchymal stromal cells: clinical challenges and therapeutic opportunities. Cell Stem Cell. 2018;22(6):824‐833. 29859173 10.1016/j.stem.2018.05.004 PMC6434696

[2] Pittenger MF , Discher DE , Peault BM , et al. Mesenchymal stem cell perspective: cell biology to clinical progress. NPJ Regen Med. 2019;4:22. 31815001 10.1038/s41536-019-0083-6 PMC6889290

[3] Lama VN , Smith L , Badri L , et al. Evidence for tissue‐resident mesenchymal stem cells in human adult lung from studies of transplanted allografts. J Clin Invest. 2007;117(4):989‐996. 17347686 10.1172/JCI29713 PMC1810571

[4] Jarvinen L , Badri L , Wettlaufer S , et al. Lung resident mesenchymal stem cells isolated from human lung allografts inhibit T cell proliferation via a soluble mediator. J Immunol. 2008;181(6):4389‐4396. 18768898 10.4049/jimmunol.181.6.4389 PMC3644960

[5] Sabatini F , Petecchia L , Tavian M , de Villeroché VJ , Rossi GA , Brouty‐Boyé D . Human bronchial fibroblasts exhibit a mesenchymal stem cell phenotype and multilineage differentiating potentialities. Lab Invest. 2005;85(8):962‐971. 15924148 10.1038/labinvest.3700300

[6] Vella S , Conaldi PG , Cova E , et al. Lung resident mesenchymal cells isolated from patients with the bronchiolitis obliterans syndrome display a deregulated epigenetic profile. Sci Rep. 2018;8(1):11167. 30042393 10.1038/s41598-018-29504-5 PMC6057887

[7] Collins JJ , Thebaud B . Lung mesenchymal stromal cells in development and disease: to serve and protect? Antioxid Redox Signal. 2014;21(13):1849‐1862. 24350665 10.1089/ars.2013.5781

[8] Chen YC , Gonzalez ME , Burman B , et al. Mesenchymal stem/stromal cell engulfment reveals metastatic advantage in breast cancer. Cell Rep. 2019;27(13):3916‐3926.e3915. 31242423 10.1016/j.celrep.2019.05.084 PMC6657699

[9] Klein D , Steens J , Wiesemann A , et al. Mesenchymal stem cell therapy protects lungs from radiation‐induced endothelial cell loss by restoring superoxide dismutase 1 expression. Antioxid Redox Signal. 2017;26(11):563‐582. 27572073 10.1089/ars.2016.6748 PMC5393411

[10] Fan XL , Zhang Y , Li X , Fu QL . Mechanisms underlying the protective effects of mesenchymal stem cell‐based therapy. Cell Mol Life Sci. 2020;77:2771‐2794. 31965214 10.1007/s00018-020-03454-6 PMC7223321

[11] Dominici M , Le Blanc K , Mueller I , et al. Minimal criteria for defining multipotent mesenchymal stromal cells. The International Society for Cellular Therapy position statement. Cytotherapy. 2006;8(4):315‐317. 16923606 10.1080/14653240600855905

[12] Viswanathan S , Shi Y , Galipeau J , et al. Mesenchymal stem versus stromal cells: International Society for Cell & Gene Therapy (ISCT(R)) Mesenchymal Stromal Cell committee position statement on nomenclature. Cytotherapy. 2019;21(10):1019‐1024. 31526643 10.1016/j.jcyt.2019.08.002

[13] Hu Y , Zhang Z , Torsney E , et al. Abundant progenitor cells in the adventitia contribute to atherosclerosis of vein grafts in ApoE‐deficient mice. J Clin Invest. 2004;113(9):1258‐1265. 15124016 10.1172/JCI19628 PMC398426

[14] Howson KM , Aplin AC , Gelati M , Alessandri G , Parati EA , Nicosia RF . The postnatal rat aorta contains pericyte progenitor cells that form spheroidal colonies in suspension culture. Am J Physiol Cell Physiol. 2005;289(6):C1396‐C1407. 16079185 10.1152/ajpcell.00168.2005

[15] Stenmark KR , Davie N , Frid M , Gerasimovskaya E , Das M . Role of the adventitia in pulmonary vascular remodeling. Physiology (Bethesda). 2006;21:134‐145. 16565479 10.1152/physiol.00053.2005

[16] Zengin E , Chalajour F , Gehling UM , et al. Vascular wall resident progenitor cells: a source for postnatal vasculogenesis. Development. 2006;133(8):1543‐1551. 16524930 10.1242/dev.02315

[17] Ergun S , Tilki D , Klein D . Vascular wall as a reservoir for different types of stem and progenitor cells. Antioxid Redox Signal. 2011;15(4):981‐995. 20712422 10.1089/ars.2010.3507

[18] Klein D , Weisshardt P , Kleff V , et al. Vascular wall‐resident CD44+ multipotent stem cells give rise to pericytes and smooth muscle cells and contribute to new vessel maturation. PLoS One. 2011;6(5):e20540. 21637782 10.1371/journal.pone.0020540 PMC3102739

[19] Lu W , Li X . Vascular stem/progenitor cells: functions and signaling pathways. Cell Mol Life Sci. 2018;75(5):859‐869. 28956069 10.1007/s00018-017-2662-2 PMC11105279

[20] Worsdorfer P , Mekala SR , Bauer J , et al. The vascular adventitia: an endogenous, omnipresent source of stem cells in the body. Pharmacol Ther. 2017;171:13‐29. 27498405 10.1016/j.pharmthera.2016.07.017

[21] Klein D . iPSCs‐based generation of vascular cells: reprogramming approaches and applications. Cell Mol Life Sci. 2018;75(8):1411‐1433. 29243171 10.1007/s00018-017-2730-7 PMC5852192

[22] Klein D , Hohn HP , Kleff V , Tilki D , Ergün S . Vascular wall‐resident stem cells. Histol Histopathol. 2010;25(5):681‐689. 20238305 10.14670/HH-25.681

[23] Wiesemann A , Ketteler J , Slama A , et al. Inhibition of radiation‐induced Ccl2 signaling protects lungs from vascular dysfunction and endothelial cell loss. Antioxid Redox Signal. 2019;30(2):213‐231. 29463096 10.1089/ars.2017.7458

[24] Klein D , Benchellal M , Kleff V , Jakob HG , Ergün S . Hox genes are involved in vascular wall‐resident multipotent stem cell differentiation into smooth muscle cells. Sci Rep. 2013;3:2178. 24145756 10.1038/srep02178 PMC3804857

[25] Steens J , Unger K , Klar L , et al. Direct conversion of human fibroblasts into therapeutically active vascular wall‐typical mesenchymal stem cells. Cell Mol Life Sci. 2020;77:3401‐3422. 31712992 10.1007/s00018-019-03358-0 PMC7426315

[26] Klein D . Improved isolation of human vascular wall‐resident mesenchymal stem cells. Methods Mol Biol. 2020;2155:71‐81. 32474868 10.1007/978-1-0716-0655-1_6

[27] Klein D , Schmetter A , Imsak R , et al. Therapy with multipotent mesenchymal stromal cells protects lungs from radiation‐induced injury and reduces the risk of lung metastasis. Antioxid Redox Signal. 2016;24(2):53‐69. 26066676 10.1089/ars.2014.6183

[28] Steens J , Zuk M , Benchellal M , et al. In vitro generation of vascular wall‐resident multipotent stem cells of mesenchymal nature from murine induced pluripotent stem cells. Stem Cell Rep. 2017;8(4):919‐932. 10.1016/j.stemcr.2017.03.001 PMC5390238 28366456

[29] Ketteler J , Wittka A , Leonetti D , et al. Caveolin‐1 regulates the ASMase/ceramide‐mediated radiation response of endothelial cells in the context of tumor‐stroma interactions. Cell Death Dis. 2020;11(4):228. 32273493 10.1038/s41419-020-2418-z PMC7145831

[30] Gyorffy B , Surowiak P , Budczies J , et al. Online survival analysis software to assess the prognostic value of biomarkers using transcriptomic data in non‐small‐cell lung cancer. PLoS One. 2013;8(12):e82241. 24367507 10.1371/journal.pone.0082241 PMC3867325

[31] Hou GX , Liu P , Yang J , Wen S . Mining expression and prognosis of topoisomerase isoforms in non‐small‐cell lung cancer by using Oncomine and Kaplan‐Meier plotter. PLoS One. 2017;12(3):e0174515. 28355294 10.1371/journal.pone.0174515 PMC5371362

[32] Crisan M , Yap S , Casteilla L , et al. A perivascular origin for mesenchymal stem cells in multiple human organs. Cell Stem Cell. 2008;3(3):301‐313. 18786417 10.1016/j.stem.2008.07.003

[33] Caplan AI . New MSC: MSCs as pericytes are sentinels and gatekeepers. J Orthop Res. 2017;35(6):1151‐1159. 28294393 10.1002/jor.23560

[34] Caplan AI . All MSCs are pericytes? Cell Stem Cell. 2008;3(3):229‐230. 18786406 10.1016/j.stem.2008.08.008

[35] Armulik A , Genove G , Betsholtz C . Pericytes: developmental, physiological, and pathological perspectives, problems, and promises. Dev Cell. 2011;21(2):193‐215. 21839917 10.1016/j.devcel.2011.07.001

[36] Diaz‐Flores L , Gutierrez R , Madrid JF , et al. Pericytes. Morphofunction, interactions and pathology in a quiescent and activated mesenchymal cell niche. Histol Histopathol. 2009;24(7):909‐969. 19475537 10.14670/HH-24.909

[37] Sims DE . The pericyte–a review. Tissue Cell. 1986;18(2):153‐174. 3085281 10.1016/0040-8166(86)90026-1

[38] Ohyama K , Matsumoto Y , Takanami K , et al. Coronary adventitial and perivascular adipose tissue inflammation in patients with vasospastic angina. J Am Coll Cardiol. 2018;71(4):414‐425. 29389358 10.1016/j.jacc.2017.11.046

[39] Falk E , Thim T , Kristensen IB . Atherosclerotic plaque, adventitia, perivascular fat, and carotid imaging. JACC Cardiovasc Imaging. 2009;2(2):183‐186. 19356554 10.1016/j.jcmg.2008.11.005

[40] Tinajero MG , Gotlieb AI . Recent developments in vascular adventitial pathobiology: the dynamic adventitia as a complex regulator of vascular disease. Am J Pathol. 2020;190(3):520‐534. 31866347 10.1016/j.ajpath.2019.10.021

[41] Klein D . Vascular Wall‐resident multipotent stem cells of mesenchymal nature within the process of vascular Remodeling: cellular basis, clinical relevance, and implications for stem cell therapy. Stem Cells Int. 2016;2016:1905846. 26880936 10.1155/2016/1905846 PMC4736960

[42] Corselli M , Chen CW , Sun B , Yap S , Rubin JP , Péault B . The tunica adventitia of human arteries and veins as a source of mesenchymal stem cells. Stem Cells Dev. 2012;21(8):1299‐1308. 21861688 10.1089/scd.2011.0200 PMC3353742

[43] Pitera JE , Smith VV , Thorogood P , Milla PJ . Coordinated expression of 3′ hox genes during murine embryonal gut development: an enteric Hox code. Gastroenterology. 1999;117(6):1339‐1351. 10579975 10.1016/s0016-5085(99)70284-2

[44] Michaut L , Jansen HJ , Bardine N , Durston AJ , Gehring WJ . Analyzing the function of a hox gene: an evolutionary approach. Dev Growth Differ. 2011;53(9):982‐993. 22150153 10.1111/j.1440-169X.2011.01307.x

[45] Montavon T , Duboule D . Chromatin organization and global regulation of Hox gene clusters. Philos Trans R Soc Lond B Biol Sci. 2013;368(1620):20120367. 23650639 10.1098/rstb.2012.0367 PMC3682730

[46] Smith J , Zyoud A , Allegrucci C . A case of identity: HOX genes in Normal and cancer stem cells. Cancers (Basel). 2019;11(4):512‐524. 10.3390/cancers11040512 PMC6521190 30974862

[47] Liedtke S , Buchheiser A , Bosch J , et al. The HOX code as a “biological fingerprint” to distinguish functionally distinct stem cell populations derived from cord blood. Stem Cell Res. 2010;5(1):40‐50. 20434420 10.1016/j.scr.2010.03.004

[48] Rolandsson Enes S , Andersson Sjoland A , Skog I , et al. MSC from fetal and adult lungs possess lung‐specific properties compared to bone marrow‐derived MSC. Sci Rep. 2016;6:29160. 27381039 10.1038/srep29160 PMC4933903

[49] Ito H , Matsuo Y , Ohtsu S , et al. Impact of histology on patterns of failure and clinical outcomes in patients treated with definitive chemoradiotherapy for locally advanced non‐small cell lung cancer. Int J Clin Oncol. 2020;25(2):274‐281. 31667664 10.1007/s10147-019-01566-z

[50] Prabavathy D , Swarnalatha Y , Ramadoss N . Lung cancer stem cells‐origin, characteristics and therapy. Stem Cell Investig. 2018;5:6. 10.21037/sci.2018.02.01 PMC5897668 29682513

[51] Smith BA , Balanis NG , Nanjundiah A , et al. A human adult stem cell signature marks aggressive variants across epithelial cancers. Cell Rep. 2018;24(12):3353‐3366.e3355. 30232014 10.1016/j.celrep.2018.08.062 PMC6382070

[52] Hardavella G , George R , Sethi T . Lung cancer stem cells‐characteristics, phenotype. Transl Lung Cancer Res. 2016;5(3):272‐279. 27413709 10.21037/tlcr.2016.02.01 PMC4931140

[53] Hanna JM , Onaitis MW . Cell of origin of lung cancer. J Carcinog. 2013;12:6. 23599688 10.4103/1477-3163.109033 PMC3622445

[54] Sutherland KD , Berns A . Cell of origin of lung cancer. Mol Oncol. 2010;4(5):397‐403. 20594926 10.1016/j.molonc.2010.05.002 PMC5527931

[55] Li F , He J , Wei J , et al. Diversity of epithelial stem cell types in adult lung. Stem Cells Int. 2015;2015:728307. 25810726 10.1155/2015/728307 PMC4354973

[56] Park E , Park SY , Sun PL , et al. Prognostic significance of stem cell‐related marker expression and its correlation with histologic subtypes in lung adenocarcinoma. Oncotarget. 2016;7(27):42502‐42512. 27285762 10.18632/oncotarget.9894 PMC5173151

[57] Visvader JE . Cells of origin in cancer. Nature. 2011;469(7330):314‐322. 21248838 10.1038/nature09781

[58] Pittenger MF , Mackay AM , Beck SC , et al. Multilineage potential of adult human mesenchymal stem cells. Science. 1999;284(5411):143‐147. 10102814 10.1126/science.284.5411.143

[59] Basil MC , Katzen J , Engler AE , et al. The cellular and physiological basis for lung repair and regeneration: past, present, and future. Cell Stem Cell. 2020;26(4):482‐502. 32243808 10.1016/j.stem.2020.03.009 PMC7128675

[60] Tiberio C , Barba P , Magli MC , et al. HOX gene expression in human small‐cell lung cancers xenografted into nude mice. Int J Cancer. 1994;58(4):608‐615. 7914516 10.1002/ijc.2910580426

[61] Flagiello D , Gibaud A , Dutrillaux B , Poupon MF , Malfoy B . Distinct patterns of all‐trans retinoic acid dependent expression of HOXB and HOXC homeogenes in human embryonal and small‐cell lung carcinoma cell lines. FEBS Lett. 1997;415(3):263‐267. 9357979 10.1016/s0014-5793(97)01118-6

[62] Monterisi S , Lo Riso P , Russo K , et al. HOXB7 overexpression in lung cancer is a hallmark of acquired stem‐like phenotype. Oncogene. 2018;37(26):3575‐3588. 29576613 10.1038/s41388-018-0229-9

[63] Yang Y , Tang X , Song X , et al. Evidence for an oncogenic role of HOXC6 in human non‐small cell lung cancer. PeerJ. 2019;7:e6629. 30993034 10.7717/peerj.6629 PMC6461029

[64] Liu H , Zhang M , Xu S , et al. HOXC8 promotes proliferation and migration through transcriptional up‐regulation of TGFbeta1 in non‐small cell lung cancer. Oncogenesis. 2018;7(2):1. 29367650 10.1038/s41389-017-0016-4 PMC5833702

[65] Javan MR , Khosrojerdi A , Moazzeni SM . New insights into implementation of mesenchymal stem cells in cancer therapy: prospects for anti‐angiogenesis treatment. Front Oncol. 2019;9:840. 31555593 10.3389/fonc.2019.00840 PMC6722482

[66] Ramasamy R , Lam EW , Soeiro I , et al. Mesenchymal stem cells inhibit proliferation and apoptosis of tumor cells: impact on in vivo tumor growth. Leukemia. 2007;21(2):304‐310. 17170725 10.1038/sj.leu.2404489

[67] Du J , Zhou L , Chen X , et al. IFN‐gamma‐primed human bone marrow mesenchymal stem cells induce tumor cell apoptosis in vitro via tumor necrosis factor‐related apoptosis‐inducing ligand. Int J Biochem Cell Biol. 2012;44(8):1305‐1314. 22554587 10.1016/j.biocel.2012.04.015

[68] Wang X , Chen H , Zeng X , et al. Efficient lung cancer‐targeted drug delivery via a nanoparticle/MSC system. Acta Pharm Sin B. 2019;9(1):167‐176. 30766788 10.1016/j.apsb.2018.08.006 PMC6362298

[69] Shi Y , Du L , Lin L , et al. Tumour‐associated mesenchymal stem/stromal cells: emerging therapeutic targets. Nat Rev Drug Discov. 2017;16(1):35‐52. 27811929 10.1038/nrd.2016.193

[70] Attar‐Schneider O , Drucker L , Gottfried M . The effect of mesenchymal stem cells' secretome on lung cancer progression is contingent on their origin: primary or metastatic niche. Lab Invest. 2018;98(12):1549‐1561. 30089856 10.1038/s41374-018-0110-z

[71] Attar‐Schneider O , Dabbah M , Drucker L , Gottfried M . Niche origin of mesenchymal stem cells derived microvesicles determines opposing effects on NSCLC: primary versus metastatic. Cell Signal. 2020;65:109456. 31672605 10.1016/j.cellsig.2019.109456

