# Supplementary material for: The vascular nature of lung‐resident mesenchymal stem cells
Source: Stem Cells Transl Med. 2020 Aug 24;10(1):128–43. doi: 10.1002/sctm.20-0191 (PMC7780817; doi:10.1002/sctm.20-0191)
Supplement: Supplementary file 1 — Data S1. Supporting Information. [file SCT3-10-128-s001.pdf]

Supplemental Information

The vascular nature of lung resident mesenchymal stem cells

Jennifer Steens[1], Lea Klar[1], Christine Hansel[1], Alexis Slama[2], Thomas Hager[3], Verena Jendrossek[1], Clemens Aigner[2], Diana Klein[1]

[1]Institute of Cell Biology (Cancer Research), University of Duisburg-Essen, University Hospital, 45122 Essen, Germany.
[2]Department of Thoracic Surgery and Surgical Endoscopy, Ruhrlandklinik-University Clinic Essen, 45239 Essen, Germany
[3]Institute of Pathology, University Clinic Essen, University of Duisburg-Essen, Essen, 45122 Germany

Supplemental Figures

Supplemental Figure S1

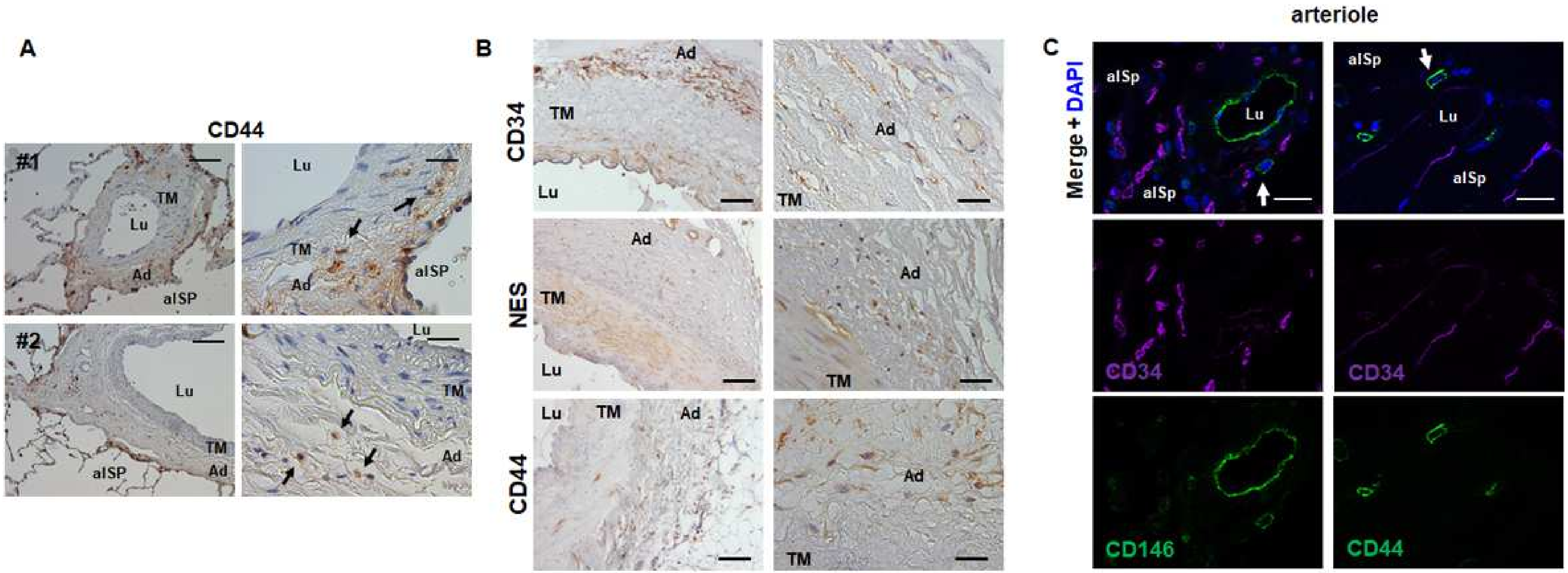

Supplemental Figure S2

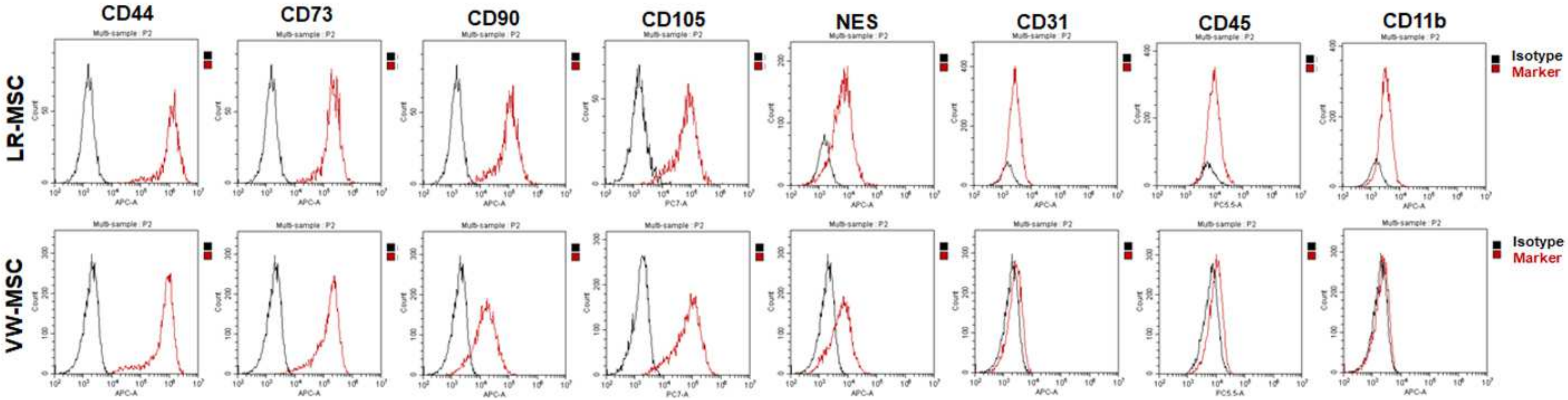

Supplemental Figure S3

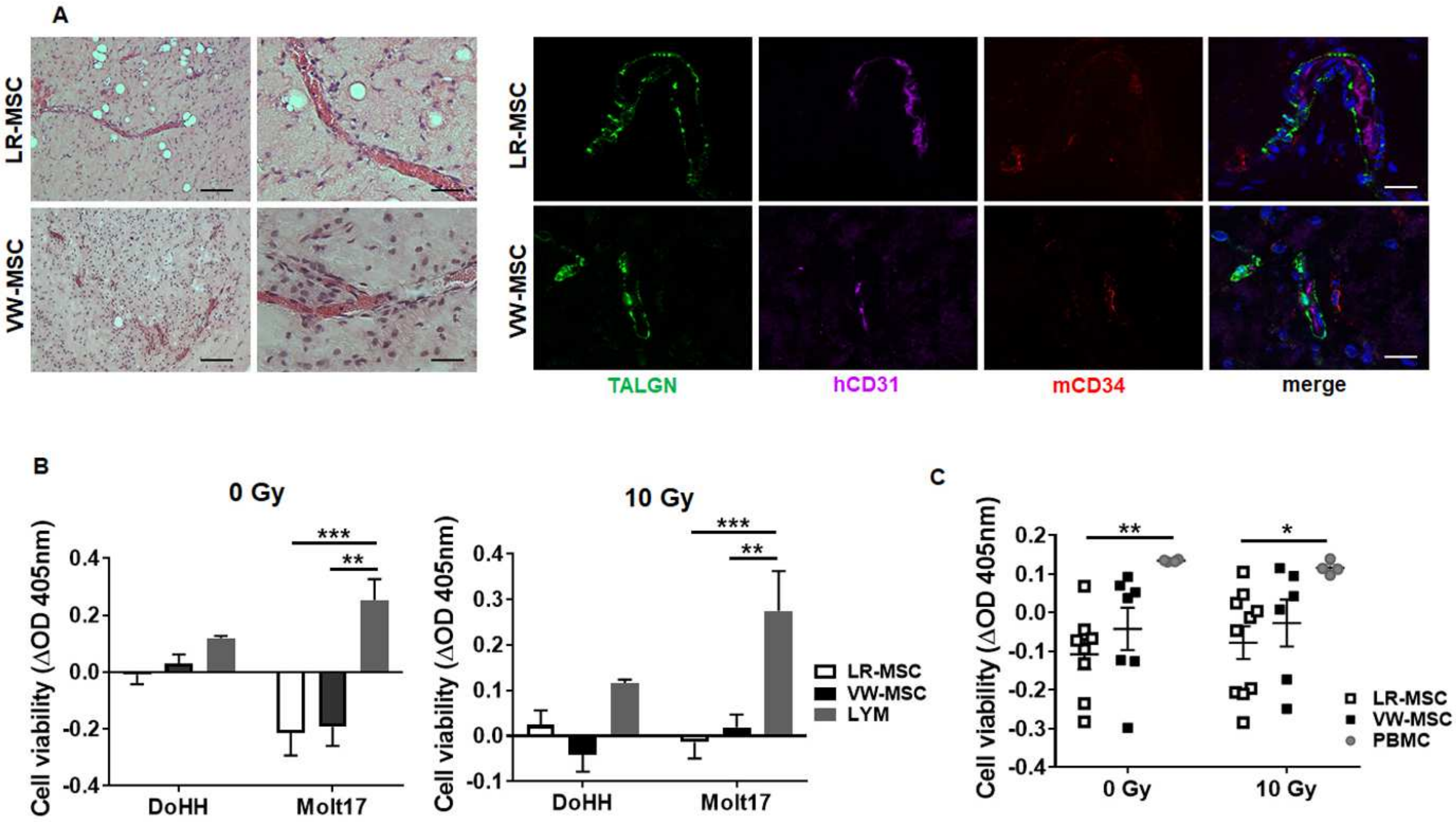

Supplemental Figure S4

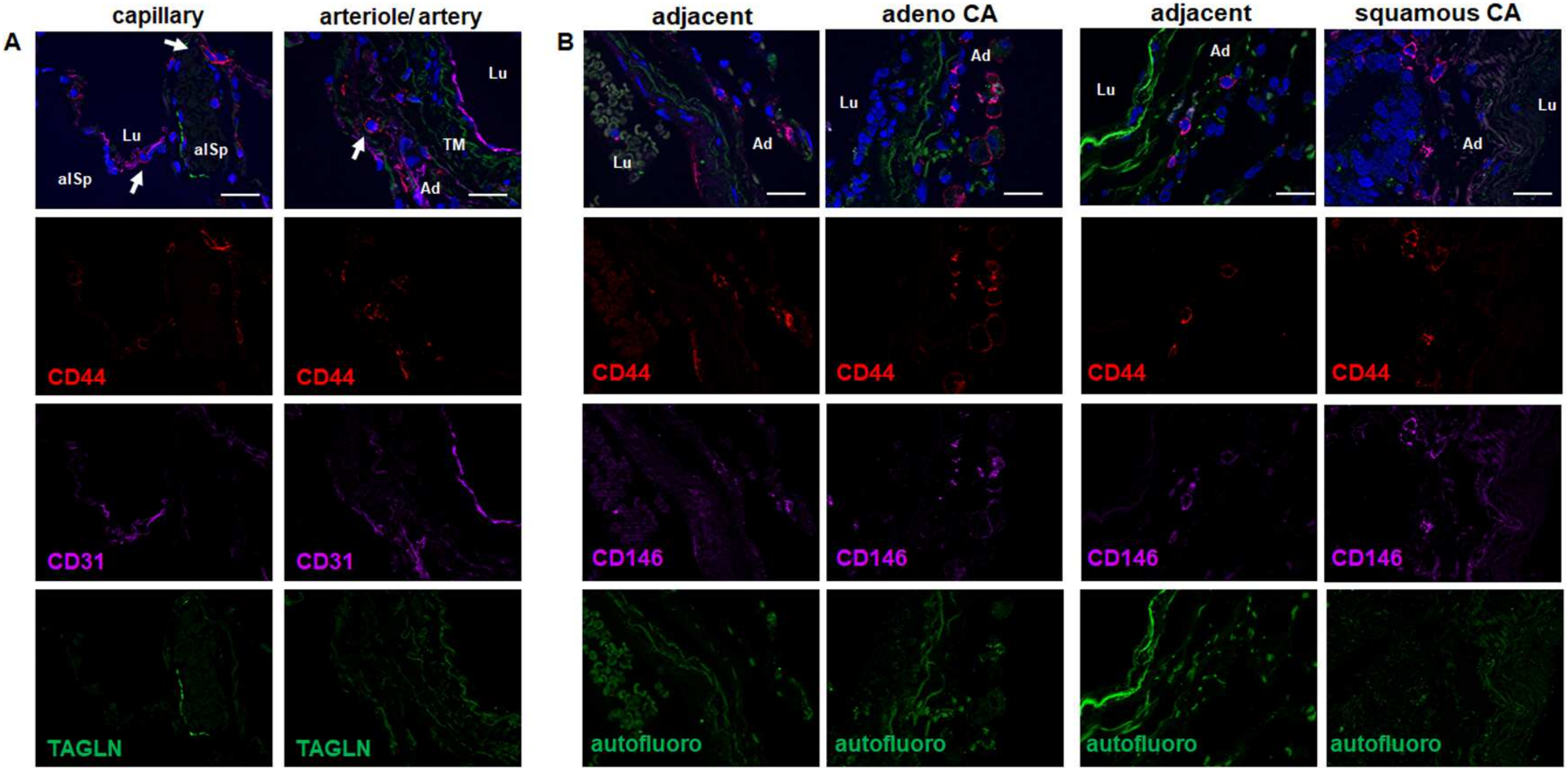

Supplemental Figure S5

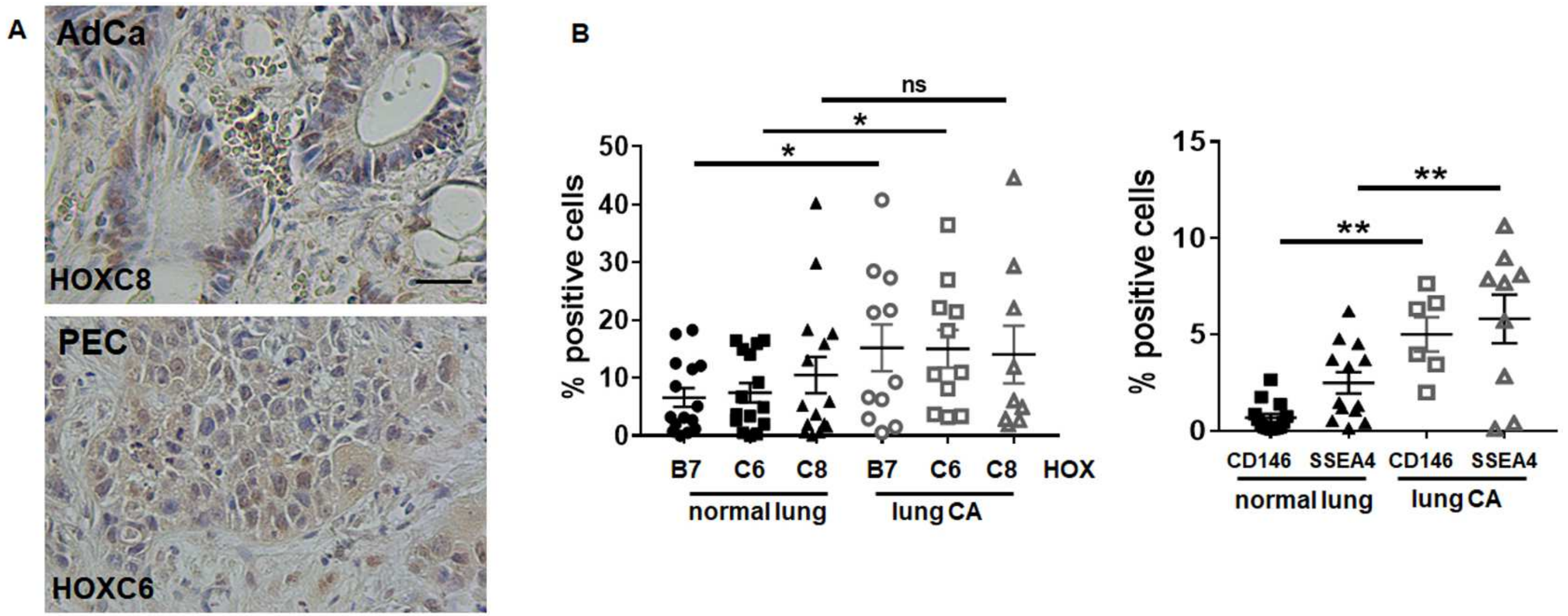

Supplemental Figure Legends

Supplemental Figure S1

Localization of vascular wall-resident putative mesenchymal stem cells

(**A**) Additional CD44 staining of normal lung tissue sections were shown to highlight the presence of putative CD44-positive mesenchymal stem cells (MSCs) (visualized by the brown color) within the vascular mural zone of large and mid-sized arteries. Scale bar indicates 100 µm (left panel) and 10 µm for the higher magnification images (right panel). (**B**) Vascular-wall-resident MSCs within the hITA wall: Immunostainings of hITA (human internal thoracic artery) sections show that the endothelial progenitor cell (EPC) marker CD34 marks the vasculogenic zone within the adventitia (Ad). In addition, a slight immunoreactivity of endothelial cells facing the vessel lumen (Lu) can be seen, as well as an increased CD34 immunoreactivity in the subendothelial space. The MSC marker nestin (NES) is detected in smooth muscle cells (SMC) of the tunica media (TM), but also in single cells of vasculogenic zone within the adventitia close to the SMC layer. CD44 positive cells are predominantly seen in the vasculogenic zone within the adventitia, near to the as highlighted by the higher magnifications (right panel). Scale bar indicates 50 µm (left panel) and 10 µm for the higher magnification images (right panel). (**C**) Co-localization of MSC marker proteins in putative MSCs of lung blood vessels (arterioles): Double-immunostainings of normal lung sections using antibodies against typical MSC marker proteins CD44 and CD146 demonstrate that CD44+ cells (putative MSCs) within the alveolar (stem cell) niche are negative for endothelial progenitor cell marker CD34. Representative lung photographs of intermediate blood vessels (arterioles) are shown. Lu lumen, TM tunica media, Ad adventitia. Scale bar indicates 10 µm.

Supplemental Figure S2

Flow cytometry analysis of MSC marker proteins of cultured (plastic-adherent) LR-MSCs as compared to VW-MSCs

Single cells suspensions were analyzed by FACS with indicated MSC marker antibodies (biological replicates: n=6-8 for each epitope and group). Representative histograms are shown.

Supplemental Figure S3

Contribution of LR- and VW-MSCs to new vessel formation *in vivo* and therapeutic potential of LR-MSCs

(**A**) LR-MSCs and VW-MSCs were grafted together with human endothelial cells (AS-M5) in Matrigel subcutaneously into NMRI nude mice for 14 days. Histological (left panel) using haematoxylin and eosin staining and immunofluorescent analyses (right panel) of isolated plug tissues were performed. Functionally perfused blood vessels within the plugs showed numerous erythrocytes (pink/red) within the vessel lumen as detected by phase contrast microscopy (left panel). Triple-staining for human CD31 (violet), mouse CD34 (red) and transgelin (TAGLN; green) showed a close assembly of TAGLN-positive cells to the vessel wall formed by the implanted endothelial cells (right panel). Within the plugs, only a minor reactivity to mouse structures (CD34) was detected. DAPI was used for nuclei staining. Scale bars: 100 µm (H&E), 50µm (fluorescence).Representative images of n=4 independent experiments are shown. (**B, C**) Verification of lymphocyte proliferation inhibition (mixed lymphocyte reaction) after prolonged co-culture as shown in Figure 3C: LR-MSCs as well as control VW-MSCs were co-cultured with different lymphoma cells (DoHH2 and MOLT17) (**B**)

or peripheral blood mononuclear cells (PBMC) isolated from healthy donors (**C**) for 96 hours. Cell-cycle arrested, irradiated (10Gy, 24 hours prior to co-culture) MSCs were used to exclude possible effects mediated by their proliferation (**B**, right diagram; **C**, right). Cell proliferation was determined using a WST-1 reagent-based tetrazolium reduction assay and related to proliferation of lymphoma (LYM) cells or PBMCs alone (biological replicates: LYM n= 3, VW-MSC n=6, LR-MSC n=9, *P* by two-way ANOVA followed by post-hoc Tukey's multiple comparisons test: ***P* ≤ 0.01; ****P* ≤ 0.005, left diagrams; PBMC n= 4, VW-MSC n=8, LR-MSC n=12, *P* by two-way ANOVA followed by post-hoc Tukey's multiple comparisons test: **P* ≤ 0.05; ***P* ≤ 0.001, right diagram) measured in quadruplets each.

Supplemental Figure S4

Co-localization of CD44-postitive vascular mural cells (LR-MSCs) of normal lung blood vessels

(**A**) Additional triple-immunofluorescent staining of normal lung tissue sections was used to highlight the presence of putative CD44-positive MSCs (red) within the vascular mural zone of larger arteries and capillaries (according to the NSCLC staining's presented in Figure 6). The SMC marker transgelin (TAGLN) is shown in green and the EPC marker CD34 in purple. Nuclei were visualized using DAPI (blue). Arrows point towards CD44-positive cells residing in the vasculogenic zone. (**B**) Double-immunofluorescent staining of normal lung tissue sections further confirmed that CD44-positive MSCs (red) within the vascular mural zone of larger arteries and capillaries co-express the MSC marker CD146 (purple). Increased excitation to enforced green fluorescence (auto-fluorescence) was used to visualize the elastic fibers. Lu lumen, TM tunica media, Ad adventitia. Representative lung photographs are shown.

Supplemental Figure S5

Higher magnification images for HOX immunohistochemistry of human NSCLC tissues and flow cytometry quantifications of putative MSCs

(**A**) Paraffin-sections of human NSCLC specimen that were stained for the indicated HOX antibodies in combination with DAB (as shown in Figure 6) were magnified in order to visualize the nuclear localization of the HOX proteins. Scale bar: 5 µm. (**B**) Normal lung as well as lung cancer tissues were homogenized by collagenase-digestion and the crude cell extract was analyzed by flow cytometry using the indicated HOX antibodies and MSC markers. Data (column scatter plots) include the mean +/- SEM, n = 6-9. *P* by one-way ANOVA followed by post-hoc Tukey's multiple comparisons test (MSC markers; ***P* ≤ 0.0) or unpaired two-tailed t-test (HOX; **P* ≤ 0.05).

Supplemental Material and Methods

Matrigel Plug Assay

Matrigel plugs were performed and collected as previously described [1,2]. In brief, NMRI nu/nu mice (Harlan Laboratories) were anesthetized by injection of intraperitoneal Rompun/Hostaket and the pre-cooled growth factor reduced (GFR)-Matrigel cell solution (200 µl/injection) containing human AS-M5 endothelial cells and VW-MSCs or LR-MSCs was injected subcutaneously. At day 14, mice were killed by isoflurane euthanasia and plugs were removed. Plug samples were fixed with 4% paraformaldehyde (PFA) and subjected for paraffin

embedding and sectioning. Mice were kept under standard conditions (12 hours light and dark cycle, food and water ad libitum) in the Central Animal Facility of the University Hospital Essen. All procedures involving mice were approved by the local institutional Animal Care Committee (Regierungspräsidium Düsseldorf 8.87-50.10.37.09.183; Az84-02.04.2012.A034; Az84-02.04.2016.A010).

Mixed lymphocyte reaction

Peripheral blood mononuclear cell (PBMC) derived from healthy donors were isolated by Ficoll-paque density centrifugation, cultured in the presence of the nonspecific mitogens phytohemagglutinin (10µg/ml) for 48 hours and co-cultured (20.000 cells per condition) with respective MSCs (5000 cells per well) for additional 96 hours. Cell proliferation was determined using the WST-1 reagent as described in Material and Methods.

Spheroid culture and CFU assay of lung cancer cells

NCI-H460 [H460] (ATCC® HTB-177™; Manassas, VA, USA) lung cancer cells were cultured in RPMI 1640 medium (Gibco, ThermoFisher, Waltham, MA, USA) supplemented with 10 % fetal calf serum (FCS) and 100 U Penicillin/Streptomycin (Sigma Aldrich, St. Louis, MO, USA) at 37°C, 5 % CO2 and 95 % humidity. All cells were routinely tested for mycoplasma contamination (every two weeks) and periodic authenticated by STR profiling (if necessary, no later than yearly). For spheroid culture, H460 cells were cultured alone or in combination with LR- and VW-MSCs in hanging drops for 24 h (ratio 1/1). Afterwards, spheroids were plated in normal growth medium (NGM) with growth-factor reduced Matrigel (Corning, NY, USA) (dilution: 1/2). Pictures were taken directly and 48 h after treatment at 10x magnification. Size was measured and calculated using ImageJ software. For the detection of cell death, spheroids were incubated thereafter for additional 15 min with propidium iodide (50 µg/mL) and DAPI (4′,6-diamidino-2-phenylindole) for nuclei staining and analyzed by fluorescent and phase contrast microscopy [3]. Irradiation of respective culture plates was performed using an Isovolt-320-X-ray machine (Seifert-Pantak) at 320 kV, 10 mA and a 1,65 mm aluminum filter at a distance of 50 cm. The dose rate was approximately 3 Gy/min with an energy of the tube of 90 kV (~ 45 keV X-rays). For CFU, H460 cells were plated (triplicates) at low densities (100-1000 cells), irradiated and subsequently incubated with supernatants/ conditioned media of LR- and VW-MSCs for additional 10 days. For conditioned media, LR- and VW-MSCs were grown until 80 % confluency in NGM, irradiated with 0 Gy (control) or 10Gy and cultured for additional 48 h in low FCS medium (2 %). Supernatant was harvested and centrifuged to separate dead cells. Conditioned medium was used with NGM (ratio 1:2).

Supplemental References

1 Klein D, Weisshardt P, Kleff V et al. Vascular wall-resident CD44+ multipotent stem cells give rise to pericytes and smooth muscle cells and contribute to new vessel maturation [Research Support, Non-U.S. Gov't] [in eng]. PloS one 2011;6(5):e20540.

2 Steens J, Unger K, Klar L et al. Direct conversion of human fibroblasts into therapeutically active vascular wall-typical mesenchymal stem cells. Cellular and molecular life sciences : CMLS 2019.

3 Ketteler J, Wittka A, Leonetti D et al. Caveolin-1 regulates the ASMase/ceramide-mediated radiation response of endothelial cells in the context of tumor-stroma interactions. Cell death & disease 2020;11(4):228.
